# Lanthanide-based metal halides prepared at room temperature by recrystallization method for X-ray imaging

**DOI:** 10.1038/s41377-025-01839-5

**Published:** 2025-05-14

**Authors:** Huwei Li, Kai Li, Zheyu Li, Xinyu Fu, Qingxing Yang, Nan Wang, Xinyu Wang, Jing Feng, Shuyan Song, Hongjie Zhang

**Affiliations:** 1 State Key Laboratory of Rare Earth Resource Utilization, Changchun Institute of Applied Chemistry, Chinese Academy of Sciences, Changchun, 130022 China; 2 State Key Laboratory of Inorganic Synthesis and Preparative Chemistry, College of Chemistry, Jilin University, Changchun, 130012 China; 3 School of Applied Chemistry and Engineering, University of Science and Technology of China, Hefei, 230026 China; 4 Department of Chemistry, Tsinghua University, Beijing, 100084 China

**Keywords:** Optical materials and structures, Optical physics

## Abstract

Lanthanide (Ln)-based metal halides with excellent luminescence properties, large Stokes shifts, and low toxicity have aroused wide attention as scintillators for X-ray imaging. However, the lack of fast and mild synthesis methods of Ln-based metal halides, as one of the technical challenges, limits their applications. Here, benefiting from the innovative selection of methanol and ethanol as the solvent and anti-solvent, respectively, a series of Cs_3_LnCl_6_ (Ln = Ce, Pr, Nd, Sm, Eu, Gd, Tb, Dy, Ho, Er, Tm, Yb, Lu) microcrystals (MCs) were prepared via the recrystallization method at room temperature for the first time. This recrystallization method could also realize large-scale production at one time and recyclable recrystallization of single-element MCs and the preparation of high-entropy five-element Cs_3_{TbDyHoErTm}_1_Cl_6_ crystals. Among these Cs_3_LnCl_6_ MCs, Cs_3_TbCl_6_ MCs with 4f → 5d absorption transition possess the highest photoluminescence quantum yield of 90.8%. Besides, under X-ray irradiation, Cs_3_TbCl_6_ MCs show a high light yield of ~51,800 photons MeV^−1^ and the as-fabricated thin films possess promising X-ray imaging ability and excellent spatial resolutions (12 lp mm^−1^). This work provides a new method for ultrafast preparing Ln-based metal halides under mild synthetic conditions and highlights their excellent potential as scintillators for X-ray imaging.

## Abstract (web summary)

Cs_3_LnCl_6_ (Ln = Ce–Lu) microcrystals were prepared via recrystallization method at room temperature. Cs_3_TbCl_6_ microcrystals exhibit high light yield and excellent imaging ability under X-ray irradiation.

## Graphical abstract

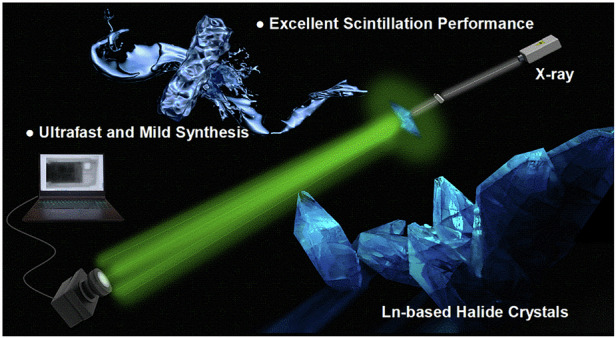

## Introduction

X-ray scintillators can convert X-ray photons to visible photons with lower energy and have been widely employed in the fields of industrial flaw detection, medical diagnosis, safety inspection, petroleum logging, environmental monitoring, etc. Recently, lead-based metal halides with large X-ray absorption coefficients, excellent photoelectric properties and solution processability have displayed remarkable scintillation performance and great promise for X-ray detection and imaging^1–7^. However, the toxicity of Pb^2+^ and self-absorption caused by small Stokes shifts inhibit their large-scale applications as scintillators. To overcome these issues, replacing Pb^2+^ with other elements to obtain lead-free metal halides with low toxicity and large Stokes shifts has captured great interest^8–12^.

Trivalent lanthanide (Ln^3+^) ions with low toxicity, large Stokes shifts, and distinct energy level transitions usually exhibit abundant and unique emissions with sharp lines in the range from ultraviolet (UV) to near-infrared (NIR) region^13–15^. Besides, quantum yield improvement, quantum cutting effect, defects passivation, multimode luminescence, etc., caused by the introduction of Ln^3+^ ions dopants, can bring lead-free metal halides excellent potentials in the applications of X-ray imaging, solid-state lighting, night vision, information storage, optical thermometry, etc^16–23^. To date, there are many reports on Ln-doped metal halides^24^, but a few on Ln-based metal halides due to the difficulty in synthesis, which limits the development of Ln-based metal halides as scintillators in the field of X-ray imaging. According to the previous reports, it is difficult to prepare Ln-based metal halides with high crystallinity by the traditional solvothermal method because Ln elements have strong oxygen affinity and hydrophilicity, and the solubility of Ln halides is quite different from other metal halides in mixed solutions^25^. Although there have been some reports of Ln-based metal halides synthesized by high-temperature solid-state synthesis or hot-injection method, such high-temperature conditions extremely limit their development^26–29^. Hence, it is necessary to develop a simple, fast, and mild synthesis method to prepare Ln-based metal halides and further explore their potential as scintillators in the field of X-ray imaging.

Herein, a series of Cs_3_LnCl_6_ (Ln = Ce, Pr, Nd, Sm, Eu, Gd, Tb, Dy, Ho, Er, Tm, Yb, Lu) metal halide microcrystals (MCs) were synthesized via a recrystallization method at room temperature. Based on this method, the feasibility of large-scale production at one time and recyclable recrystallization of single-element MCs, and the preparation of related high-entropy Ln-based metal halide crystals were investigated. Density functional theory (DFT) calculations were adopted to explore the 4f → 5d transitions or Cl → Ln charge transfer transitions in parts of Cs_3_LnCl_6_ MCs, which could overcome 4 f → 4 f parity-forbidden transitions and bring them better absorption ability in the near UV region and great PL performance. Subsequently, Cs_3_TbCl_6_, with the highest photoluminescence quantum yield (PLQY) of 90.8% among these Cs_3_LnCl_6_, was selected to investigate the potential in the application of X-ray imaging. Under X-ray irradiation, Cs_3_TbCl_6_ MCs powder with excellent X-ray scintillation performance was combined with polydimethylsiloxane (PDMS) to fabricate related thin films, which displayed great X-ray imaging ability.

## Results

**Fig. 1:**
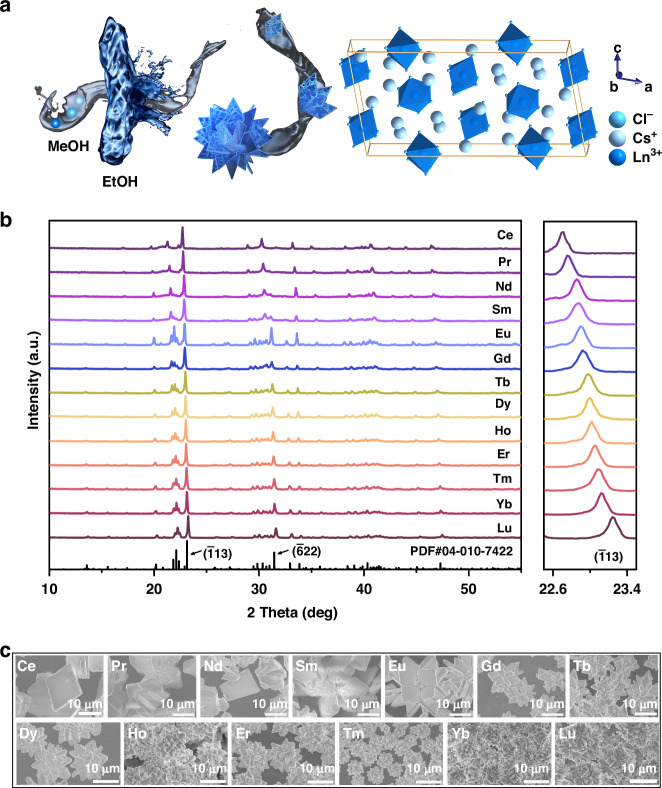
**Structure characterizations of as-prepared Cs**_**3**_**LnCl**_**6**_**. a** The schematic illustration of the synthesis procedure and crystal unit cell structure of Cs_3_LnCl_6_ MCs. **b** The powder XRD patterns and (**c**) SEM images of Cs_3_LnCl_6_ MCs

Considering the radioactivity of Pm^3+^, Ln^3+^ ions involved in this work do not include Pm^3+^. All the samples were prepared through a simple synthetic route at room temperature. As illustrated in Fig. 1a, CsCl and LnCl_3_·*x*H_2_O were dissolved in methanol (MeOH) under ultrasound, and then Cs_3_LnCl_6_ (Ln = Ce, Pr, Nd, Sm, Eu, Gd, Tb, Dy, Ho, Er, Tm, Yb, Lu) metal halides MCs can be obtained quickly within 2 min by introducing moderate ethanol (EtOH) into the mixed MeOH solution to precipitate them as powder. To prepare Cs_3_LaCl_6_, it requires the addition of cyclohexane as an anti-solvent after adding EtOH. However, the obtained metal halide is Cs_3_LaCl_6_·3H_2_O rather than Cs_3_LaCl_6_. The structural and morphological characterizations of as-prepared Cs_3_LaCl_6_·3H_2_O are shown in Supplementary Fig. S1. Therefore, in subsequent discussions, we will focus on the remaining thirteen Cs_3_LnCl_6_ MCs (Ln = Ce, Pr, Nd, Sm, Eu, Gd, Tb, Dy, Ho, Er, Tm, Yb, Lu).

The X-ray diffraction (XRD) patterns of the prepared Cs_3_LnCl_6_ MCs are displayed in Fig. 1b, Supplementary Fig. S2–9 and S10–14. Cs_3_LnCl_6_ MCs exhibit similar diffraction patterns, suggesting these MCs share the same crystal structure and space group. Specifically, the diffraction patterns of Cs_3_CeCl_6_, Cs_3_PrCl_6_, Cs_3_NdCl_6_, Cs_3_SmCl_6_, Cs_3_EuCl_6_, Cs_3_GdCl_6_, Cs_3_TbCl_6_, and Cs_3_ErCl_6_ match well with the standard patterns (PDF#04-007-9649, 04-007-9650, 04-007-9651, 04-007-9652, 04-007-9653, 04-007-9654, 04-006-9440, 04-010-7422), conforming to monoclinic crystal structure (*C2*/*c* space group) without any secondary phases (Supplementary Figs. S2–9). Nevertheless, the XRD patterns of Cs_3_DyCl_6_, Cs_3_HoCl_6_, Cs_3_TmCl_6_, Cs_3_YbCl_6_, and Cs_3_LuCl_6_ are not filed in the PDF or ICSD database. Hence, the Rietveld refinements of their diffraction patterns were performed. As shown in Supplementary Fig. S10–14 and Table S1, the Rietveld refinement X-ray diffraction plots and structural parameters of Cs_3_DyCl_6_, Cs_3_HoCl_6_, Cs_3_TmCl_6_, Cs_3_YbCl_6_, and Cs_3_LuCl_6_ were provided, and the results demonstrate their monoclinic crystal structure (*C2/c* space group) without any secondary phases. Therefore, the crystal structure of all Cs_3_LnCl_6_ MCs adopts a monoclinic *C2/c* space group (#15) (Fig. 1a), consisting of a 0D framework of spatially independent octahedra [LnCl_6_]^3-^, which are completely separated by surrounding Cs^+^ ions^27^. Meantime, from Cs_3_CeCl_6_ to Cs_3_LuCl_6_, the XRD peak ($\bar{1}$13) slightly shifts to a larger angle. This progressive shrinking of the lattice can be attributed to the gradual reduction of ionic radius from Ce^3+^ to Lu^3+^. Compared with the XRD patterns of other Cs_3_LnCl_6_, the relative intensity between parts of the XRD peaks changes in Cs_3_CeCl_6_ for the crystals could grow selectively along with different crystal planes. As displayed in the scanning electron microscopy (SEM) images (Fig. 1c), from Cs_3_CeCl_6_ to Cs_3_LuCl_6_, their morphologies transform from thin plate shape to flower shape, which implies the different crystal growth processes, possibly caused by the increasing rate of crystallization. Subsequently, the crystallization rates of these Cs_3_LnCl_6_ MCs are reflected by the productivities after adding EtOH for 60 seconds. As presented in Supplementary Fig. S15, the crystallization rates become faster and faster from Cs_3_CeCl_6_ to Cs_3_EuCl_6_, and then almost identical from Cs_3_EuCl_6_ to Cs_3_LuCl_6_, which matches well with morphological change in SEM images^30^.

**Fig. 2:**
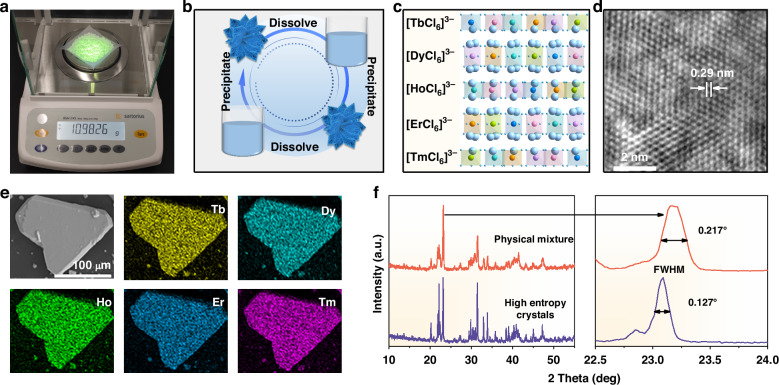
**Structure characterizations of as-prepared high entropy Cs**_**3**_**{TbDyHoErTm}**_**1**_**Cl**_**6**_**. a** Representative photograph of large-scale synthetic crystals of Cs_3_TbCl_6_ under 365 nm UV light irradiation. **b** Schematic diagram of the reversible synthesis of Cs_3_TbCl_6_ crystals. **c** The schematic illustration of high-entropy Cs_3_{TbDyHoErTm}_1_Cl_6_ crystals. The (**d**) HRTEM image and (**e**) SEM elemental mappings of high-entropy Cs_3_{TbDyHoErTm}_1_Cl_6_ crystals. **f** The general XRD and fine scans over the ($\bar{1}$13) reflection of high entropy Cs_3_{TbDyHoErTm}_1_Cl_6_ crystals and the related physical mixture of Cs_3_TbCl_6_, Cs_3_DyCl_6_, Cs_3_HoCl_6_, Cs_3_ErCl_6_, and Cs_3_TmCl_6_

In addition, this recrystallization method could also realize large-scale production at one time and excellent recyclability of single-element Cs_3_LnCl_6_ MCs and the preparation of high-entropy five-element Ln-based metal halide crystals. As shown in Fig. 2a, large quantities of Cs_3_TbCl_6_ MCs (~11.0 g) can be easily obtained at one time by enlarging metal salts in equal proportions by a factor of 100 and dissolving them in MeOH, followed by adding anti-solvent EtOH. Subsequently, the recyclability of as-prepared MCs was explored to avoid the waste of resources after completing a specific application mission. An appropriate amount of MeOH was employed to recover as-prepared Cs_3_TbCl_6_ MCs. Then, Cs_3_TbCl_6_ MCs could be precipitated again with sufficient EtOH as an anti-solvent. As shown in Fig. 2b and Supplementary Fig. S16, Cs_3_TbCl_6_ MCs could be utilized and recycled repeatedly. In addition, based on this recrystallization method, high-entropy five-element Ln-based metal halide crystals could be prepared successfully. High-entropy materials, as excellent functional materials, have attracted increasing attention, however, high temperature (~1000 °C) is generally necessary in synthetic procedures that limits their development^31,32^. Here, instead of being indirectly added, EtOH as anti-solvent was diffused slowly into the mixed MeOH solution, including metal (Cs^+^, Tb^3+^, Dy^3+^, Ho^3+^, Er^3+^, Tm^3+^) salts. After standing for 12 hours, five-element Cs_3_{TbDyHoErTm}_1_Cl_6_ metal halide crystals were successfully acquired at room temperature (Fig. 2c). High-resolution transmission electron microscopy (HRTEM) exhibits distinct lattice fringes with a lattice spacing of 0.29 nm that is indexed as crystal plane ($\bar{6}$22) of the Cs_3_{TbDyHoErTm}_1_Cl_6_ crystals phase (Fig. 2d). SEM elemental mappings reveal homogeneous distribution of all five incorporated Ln^3+^ ions within five-element Cs_3_{TbDyHoErTm}_1_Cl_6_ crystals (Fig. 2e). In Supplementary Table S2, inductively coupled plasma optical emission spectrometry (ICP-OES) provided the actual molar ratio of the five Ln^3+^ ions (at 17-25%). The XRD pattern of as-prepared five-element Cs_3_{TbDyHoErTm}_1_Cl_6_ crystals displays a similar monoclinic structure with the physical mixture of five corresponding single-element crystals (Fig. 2f). Moreover, after fine scanning the primary characteristic diffraction peak, no peak splitting happens and the full width at half maximum (FWHM) displays a smaller value compared with that of the physical mixture with the multi-phase structure, implying the single-phase structure of Cs_3_{TbDyHoErTm}_1_Cl_6_ crystals. As a result, as-prepared Cs_3_{TbDyHoErTm}_1_Cl_6_ crystals could be confirmed as single-phase high-entropy crystals, indicating that high-entropy Ln-based metal halide crystals could be successfully prepared at room temperature based on this recrystallization method.

**Fig. 3:**
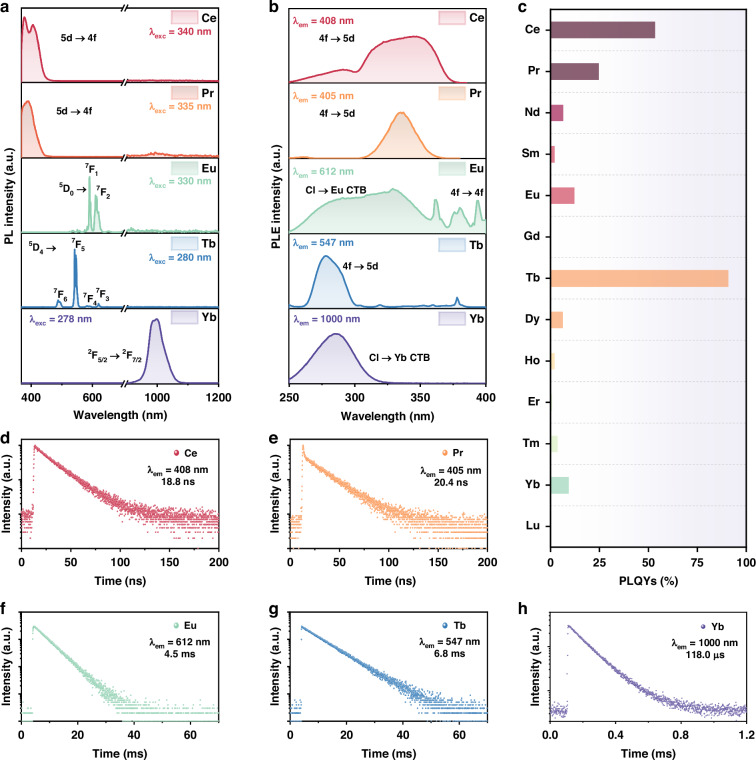
**Optical characterizations of Cs**_**3**_**LnCl**_**6**_**. a** The PL and (**b**) PLE spectra of Cs_3_CeCl_6_, Cs_3_PrCl_6_, Cs_3_EuCl_6_, Cs_3_TbCl_6_, and Cs_3_YbCl_6_ MCs. **c** The PLQYs of all Cs_3_LnCl_6_ MCs. The PL decay curves of (**d**) Cs_3_CeCl_6_, (**e**) Cs_3_PrCl_6_, (**f**) Cs_3_EuCl_6_, (**g**) Cs_3_TbCl_6_, and (**h**) Cs_3_YbCl_6_ MCs

To investigate the optical properties of as-prepared Cs_3_LnCl_6_ MCs, the absorption spectra are carried out (Supplementary Fig. S17). The absorption bands located at 340 nm for Cs_3_CeCl_6_ and 285 nm for Cs_3_TbCl_6_ could be ascribed to 4f → 5d transitions of Ce^3+^ and Tb^3+^ ions, respectively. In the absorption spectrum of Cs_3_PrCl_6_, there are a weak broadband (300–420 nm) and sharp peaks (420–600 nm), which could be attributed to 4f → 5d and 4f → 4f transitions of Pr^3+^ ions, respectively. For Cs_3_EuCl_6_ and Cs_3_YbCl_6_, the broad absorption bands are charge transfer bands (CTB) in [EuCl_6_]^3^^−^ and [YbCl_6_]^3^^−^ octahedra. While for other Cs_3_LnCl_6_, there are mainly sharp peaks from 4f → 4f transitions of Ln^3+^ ions or no obvious absorption peaks. The parity forbidden 4f → 4f transitions of Cs_3_LnCl_6_ MCs may limit their photoluminescence (PL) performance and optoelectronic applications. Then, PL spectra of these Cs_3_LnCl_6_ MCs under the excitations of specific wavelengths are shown in Fig. 3a, Supplementary Figs. S18 and S19. Among these MCs, Cs_3_CeCl_6_, Cs_3_PrCl_6_, Cs_3_TbCl_6_, and Cs_3_EuCl_6_ possess the strongest visible emissions and emit bright blue (around 400 nm; Cs_3_CeCl_6_ and Cs_3_PrCl_6_), green (547 nm; Cs_3_TbCl_6_), and red (592 nm and 612 nm; Cs_3_EuCl_6_) emissions, respectively. In the NIR range, Cs_3_YbCl_6_ MCs display the strongest emission at ~1000 nm. While for other Cs_3_LnCl_6_, only weak Ln^3+^ characteristic emissions or host emissions could be observed. The PLE spectra of Cs_3_LnCl_6_ MCs monitored at the position of Ln^3+^ characteristic emissions are shown in Fig. 3b and Supplementary Fig. S20, the excitation bands display similar patterns with their absorption spectra. Then, under the excitation of specific wavelength, absolute PLQYs of these Cs_3_LnCl_6_ MCs could be obtained (Fig. 3c and Supplementary Table S3). Among them, Cs_3_TbCl_6_ possesses the highest PLQY of 90.8%, exceeding most lead-free metal halides (Supplementary Table S4)^33–39^. From the values of PLQYs, among all Cs_3_LnCl_6_ MCs, those Cs_3_LnCl_6_ (Ln = Ce, Pr, Eu, Tb, Yb) with great absorption ability usually possess great PL performance as well. In Fig. 3d–h and Supplementary Fig. S21, the PL decay curves of Ln^3+^ characteristic emissions or host emissions in Cs_3_LnCl_6_ could provide their lifetimes that match the characteristics of the lifetimes of 5d/4f → 4f transitions of Ln^3+^ ions.

**Fig. 4:**
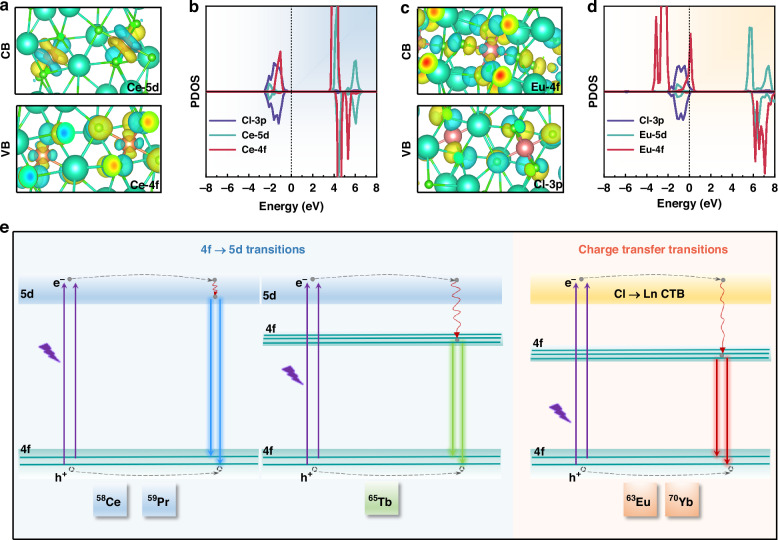
**PL mechanisms of Cs**_**3**_**LnCl**_**6**_. The relevant partial charge density maps for CB (top) and VB (bottom) regions of (**a**) Cs_3_CeCl_6_ and (**c**) Cs_3_EuCl_6_. The PDOS of (**b**) Cs_3_CeCl_6_ and (**d**) Cs_3_EuCl_6_. **e** Schematic diagram of the proposed transition mechanisms of Cs_3_LnCl_6_ (Ln = Ce, Pr, Tb, Eu, and Yb)

To further investigate the great absorption abilities in parts of Cs_3_LnCl_6_. The electronic structures of Cs_3_LnCl_6_ were investigated by theory calculation. The partial charge density maps and partial density of states (PDOS) of Cs_3_CeCl_6_ and Cs_3_EuCl_6_ were carried out for Ce^3+^ and Eu^3+^ act as typical Ln^3+^ ions to display 4f → 5d transition and Cl → Ln charge transfer transition, respectively (Fig. 4a–d). As disclosed in the partial charge density maps and PDOS of Cs_3_CeCl_6_, the valence band (VB) was composed of Cl-3p and Ce-4f-occupied orbitals. While the conduction band (CB) was composed of Ce-5d and Ce-4f empty orbitals. For comparison, the PDOS of Cs_3_LnCl_6_ (Ln = Pr, Nd, Sm, and Gd) are also calculated (Supplementary Fig. S22). It is found that the 4f occupied orbitals are much closer to 5d empty orbitals in Cs_3_CeCl_6_, which could be responsible for their greater possibility for 4f → 5d transitions. For Cs_3_EuCl_6_, the VB and CB are contributed by Cl-3p occupied orbitals and Eu-4f empty orbitals, respectively. Similarly, the Eu-4f empty orbitals are much closer to Cl-3p occupied orbitals in Cs_3_EuCl_6_, implying a higher possibility for Cl → Eu charge transfer transitions.

Tb^3+^ and Pr^3+^ ions could display 4f → 5d transitions, and Yb^3+^ ions possess Cl → Yb charge transfer transition as well, which may be attributed to the similar energy level conditions with that of Cs_3_CeCl_6_ and Cs_3_EuCl_6_, respectively. In Fig. 4e, the possible PL mechanisms of these Cs_3_LnCl_6_ (Ln = Ce, Pr, Tb, Eu, and Yb) with 4f → 5d transitions or charge transfer transitions are proposed. For Cs_3_CeCl_6_ and Cs_3_PrCl_6_, the electrons in the 4f ground states are excited to the 5 d excited states and then relaxed to the 4f ground states, giving out blue emissions due to 5d → 4f radiative recombination. For Cs_3_TbCl_6_, after being excited from 4f ground state to the 5d excited state, the electrons are nonradiatively relaxed to the Tb^3+^ 4f excited states and further relaxed to 4f ground states along with Tb^3+^ characteristic emissions from 4f → 4f transitions. While for Cs_3_EuCl_6_ and Cs_3_YbCl_6_, after being excited from Ln^3+^ 4f ground states to charge transfer states, the electrons are relaxed to Ln^3+^ 4f excited states and then relaxed to 4f ground states, resulting in corresponding Ln^3+^ emissions for radiative recombination of their 4f → 4f transitions.

For other Cs_3_LnCl_6_ without 4f → 5d transitions or charge transfer transitions, their 4f occupied orbitals may be far away from 5 d empty orbitals, and their 4f empty orbitals may be far from Cl-3p occupied orbitals, resulting in higher energy required for 4f → 5d transitions and Cl → Ln charge transfer transitions, thus decreasing the probability for these transitions under the excitation of near UV light. Therefore, it is difficult for these Cs_3_LnCl_6_ to exhibit great absorption ability based on 4f → 5d transitions or Cl → Ln charge transfer transitions. Hence, among all Cs_3_LnCl_6_, small energy level intervals from Ln-4f occupied orbitals to Ln-5d empty orbitals or from Cl-3p occupied orbitals to Ln-4f empty orbitals could result in great possibilities for 4f → 5d transitions or Cl → Ln charge transfer transitions that replace the parity forbidden 4f → 4f transitions and could be responsible for their great PL performance.

The thermogravimetric (TG) curves (Supplementary Fig. S23) show no significant weight loss (remaining > 94%) until 600 °C for these Cs_3_LnCl_6_ MCs, indicating their excellent structural stability. In Supplementary Figs. S24 and S25, the PL intensities of Cs_3_LnCl_6_ MCs remain above 88% of their initial values, and XRD patterns remain essentially unchanged after the MCs were left in a sealed environment without light exposure for 300 days, indicating the great air stability of these Cs_3_LnCl_6_ MCs.

**Fig. 5:**
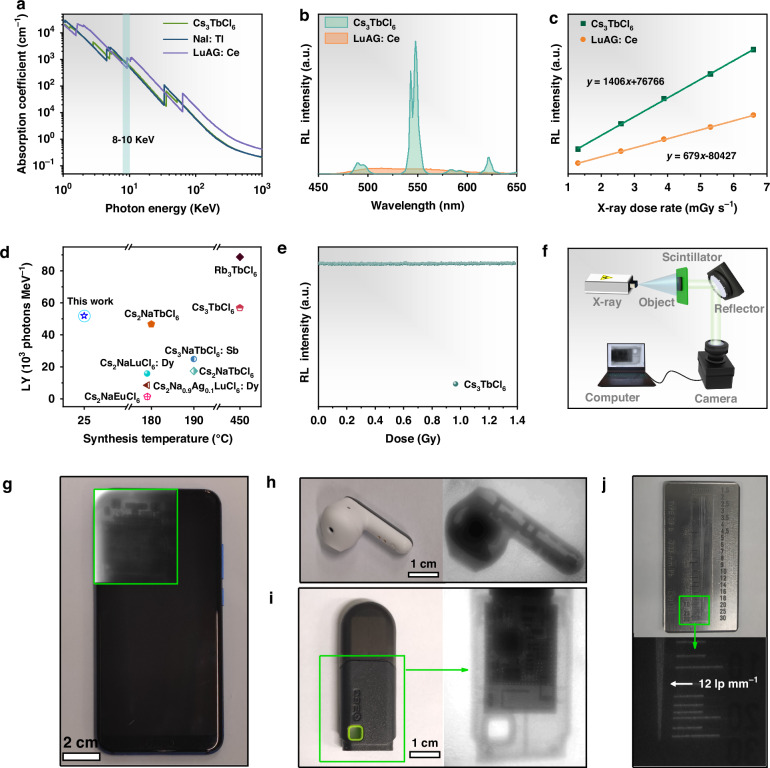
**Application of Cs**_**3**_**TbCl**_**6**_ **in X-ray imaging. a** The absorption coefficients of Cs_3_TbCl_6_ MCs, NaI:Tl and LuAG:Ce as a function of photon energy from 1 KeV to 1000 KeV. **b** RL spectra of Cs_3_TbCl_6_ and LuAG:Ce at dose rate of 6.6 mGy s^−1^. **c** RL integral intensity of Cs_3_TbCl_6_ and LuAG:Ce as a function of X-ray dose rate. **d** Comparison of LY and synthesis temperature of Cs_3_TbCl_6_ MCs in this work with the reported Ln-based metal halides scintillators. **e** The RL intensity of Cs_3_TbCl_6_ MCs as a function of cumulative dose. **f** Schematic of the X-ray imaging system. The photographs of (**g**) a smartphone, (**h**) a headset, and (**i**) a wireless network interface controller under the visible light and X-ray at a dose rate of 1.2 mGy s^−^^1^. **j** The photograph of the standard line-pair card under the visible light (top) and the corresponding X-ray image of the standard line-pair card (bottom)

The light yield (LY), as one of the important indicators for evaluating the scintillator performance, is proportional to the PLQYs of scintillators. Among these Cs_3_LnCl_6_, Cs_3_TbCl_6_ MCs with the highest PLQY and large Stokes shift could be very promising to exhibit great scintillator performance and have great potential for X-ray imaging^40^. Therefore, the X-ray scintillation performance of Cs_3_TbCl_6_ MCs was explored. The band gap of Cs_3_TbCl_6_ was calculated as 4.3 eV in Supplementary Fig. S26. Compared with commercially available scintillators, Cs_3_TbCl_6_ MCs exhibit a higher absorption coefficient than that of NaI:Tl and the equivalent absorption coefficient with that of Lu_3_Al_5_O_12_:Ce (LuAG:Ce) at 8–10 keV (Fig. 5a). For quantifying the X-ray LY of Cs_3_TbCl_6_ MCs, commercially available scintillator LuAG:Ce (with the thickness of 1 mm; the LY of 25,000 photons MeV^−1^) was selected as the reference^40,41^. Meantime, as shown in Supplementary Fig. S27a, Cs_3_TbCl_6_ sample with the thickness of 0.5 mm was prepared to unify the absorbed X-ray energy with LuAG:Ce sample. Radioluminescence (RL) spectra of Cs_3_TbCl_6_ MCs and LuAG:Ce are presented in Fig. 5b, similar Tb^3+^ characteristic emissions of Cs_3_TbCl_6_ imply the same radiative recombination channel with that under the excitation of UV light. Besides, as the X-ray dose rate increases, the RL integral intensity of Cs_3_TbCl_6_ MCs displays a linear increasing response curve (Supplementary Fig. S27b). As exhibited in Fig. 5c, the response of Cs_3_TbCl_6_ MCs is 2.07 times higher than that of LuAG:Ce and the outstanding LY of Cs_3_TbCl_6_ MCs is ~51,800 photons MeV^−1^. Compared with other Ln-based metal halide scintillators obtained via solid-state synthesis, hydrothermal synthesis or hot-injection methods in previous reports, as-prepared Cs_3_TbCl_6_ MCs could not only be synthesized quickly under mild synthetic conditions but also possess excellent LY of X-ray scintillator (Fig. 5d and Supplementary Table S5)^22,28,33,42^. Meantime, as shown in Supplementary Fig. S27c, the LY of Cs_3_TbCl_6_ MCs exceeds that of parts of commercial scintillation crystals^43–45^. Moreover, compared with commercial scintillation crystals Gd_2_O_2_S:Ce,Pr,F (LY = 35000 photons MeV^−1^), Cs_3_TbCl_6_ MCs with higher LY have great potential to replace Gd_2_O_2_S:Ce,Pr,F and be used as the next generation sensitization screen in the field of X-ray computed tomography imaging. In Supplementary Fig. S27d, when the signal-to-noise ratio (SNR) is 3, the detection limit of Cs_3_TbCl_6_ MCs is 63 nGy s^−1^, which is 87.3 times lower than those required for medical X-ray diagnosis standards (5.5 μGy s^−1^)^46^. Furthermore, under the X-ray irradiation with a cumulative dose of 1.38 Gy, the RL intensity remains unchanged, demonstrating the robust radiation hardness of Cs_3_TbCl_6_ MCs as X-ray scintillators (Fig. 5e). Subsequently, for X-ray imaging, flexible scintillation thin film (50 mm × 50 mm × 1 mm) based on Cs_3_TbCl_6_ MCs powder was prepared via blending the sample with polydimethylsiloxane (PDMS) and put it in X-ray imaging system (Fig. 5f and Supplementary Fig. S27e). As shown in Fig. 5g–i, a smartphone, a headset, and a wireless network interface controller were utilized as target objects to research the X-ray imaging ability of Cs_3_TbCl_6_@PDMS film. Under X-ray illumination, the distinct inside structure can be distinguished, indicating the realization of non-destructive testing for internal electronic components in these target objects. As presented in line-pair card imaging in Fig. 5j, the spatial resolution is derived as 12 lp mm^−1^, exceeding that of most scintillator thin films based on Ln-based metal halides in the previous reports (Supplementary Table S6)^22,27,28,42^. It is suggested that Cs_3_TbCl_6_ MCs synthesized by the recrystallization method possess excellent scintillator performance and have great potential for X-ray imaging.

## Discussion

In summary, we have prepared a series of Cs_3_LnCl_6_ MCs through a facile recrystallization method at room temperature for the first time. Especially, based on this method, large-scale production at one time and multiple recyclable recrystallizations of single-element Cs_3_LnCl_6_ MCs and the preparation of high-entropy five-element Cs_3_{TbDyHoErTm}_1_Cl_6_ metal halide crystals could also be realized. Based on DFT calculations, low energy for 4f → 5d transitions or Cl → Ln charge transfer transitions could overcome the 4f → 4f parity forbidden transitions of Ln^3+^ and bring excellent absorption ability and great PL performance of Cs_3_LnCl_6_ MCs. Especially, Cs_3_TbCl_6_ with 4f → 5d transitions absorption band possesses the highest PLQY of 90.8% among these Cs_3_LnCl_6_ MCs. Under X-ray irradiation, Cs_3_TbCl_6_ MCs show excellent X-ray scintillation performance with a high LY of ~51800 photons MeV^-1^ and the as-fabricated Cs_3_TbCl_6_@PDMS thin films possess promising X-ray imaging ability and preferable spatial resolution (12 lp mm^−1^). This work displays a novel recrystallization method for ultrafast and mild preparation of Ln-based metal halides and highlights their excellent potential as scintillators for X-ray imaging.

## Materials and methods

### Chemicals

Cesium chloride (CsCl, 99.99%), Cerium chloride heptahydrate (CeCl_3_·7H_2_O, 99%), praseodymium chloride hydrate (PrCl_3_·xH_2_O, 99.9%), neodymium chloride (NdCl_3_, 99.9%), samarium chloride hydrate (SmCl_3_·*x*H_2_O, 99.99%), Europium chloride hydrate (EuCl_3_·*x*H_2_O, 99.9%), gadolinium chloride hexahydrate (GdCl_3_·6H_2_O, 99.9%), terbium chloride hexahydrate (TbCl_3_·6H_2_O, 99.99%), dysprosium chloride hydrate (DyCl_3_·*x*H_2_O, 99.99%), holmium chloride hexahydrate (HoCl_3_·6H_2_O, 99.9%), erbium chloride hydrate (ErCl_3_·*x*H_2_O, 99.99%), thulium chloride hydrate (TmCl_3_·*x*H_2_O, 99.9%), ytterbium chloride hydrate (YbCl_3_·*x*H_2_O, 99.9%), lutetium chloride hexahydrate (LuCl_3_·6H_2_O, 99.9%) were purchased from Alfa Aesar. Methanol (MeOH, AR) and ethanol (EtOH, AR) were purchased from XiLONG SCIENCE. Polydimethylsiloxane (PDMS, SYLGARD® 184) was purchased from Dow Corning. All the chemicals were commercially purchased and used without further purification.

### Preparation of Cs_3_LnCl_6_ MCs

0.6 mmol CsCl was first dissolved in 4 mL of methanol under ultrasound. 0.2 mmol LnCl_3_·xH_2_O was dissolved in 1 mL of methanol. Then, the above two solutions were mixed evenly. After introducing 5 mL of ethanol into the mixture, white precipitate formed immediately. After standing for 2 min, the supernatant was discarded, the collected precipitation was dried at 60 °C for 10 min to obtain Cs_3_LnCl_6_ MCs.

### Preparation of Cs_3_{TbDyHoErTm}_1_Cl_6_ high entropy crystals

0.6 mmol CsCl was first dissolved in 4 mL of methanol under ultrasound. 0.04 mmol TbCl_3_·6H_2_O, 0.04 mmol DyCl_3_·*x*H_2_O, 0.04 mmol HoCl_3_·6H_2_O, 0.04 mmol ErCl_3_·xH_2_O and 0.04 mmol TmCl_3_·xH_2_O were dissolved in 1 mL of methanol. Then, the above two solutions were mixed evenly. Subsequently, an appropriate amount of ethanol was slowly diffused into the methanol solution for 12 h to form the transparent white crystals. Then, the supernatant was discarded, and the collected precipitation was dried at 60 °C for 10 min to obtain Cs_3_{TbDyHoErTm}_1_Cl_6_ high entropy crystals.

### Preparation of Cs_3_TbCl_6_@PDMS flexible thin film

First, 2.270 g of PDMS base resin and 0.2300 g of curing agent were mixed in a beaker. Then, 0.1880 g of Cs_3_TbCl_6_ powder was dispersed in the above PDMS precursor with stirring for 30 min. After curing at 100 °C for 60 min, the Cs_3_TbCl_6_@PDMS flexible thin film was obtained.

## Supplementary information

Supporting Information

## Data Availability

Data will be available when the paper is officially published.
