# Supplementary material for: Lanthanide-based metal halides prepared at room temperature by recrystallization method for X-ray imaging
Source: Light Sci Appl. 2025 May 14;14:195. doi: 10.1038/s41377-025-01839-5 (PMC12078671; doi:10.1038/s41377-025-01839-5)
Supplement: Supplementary file 1 — Supporting Information [file 41377_2025_1839_MOESM1_ESM.docx]

**Supplementary Information for**

**Lanthanide-based metal halides prepared at room temperature by recrystallization method for X-ray imaging**

Huwei Li^1,2^, Kai Li^1^, Zheyu Li^1,3^, Xinyu Fu^1,3^, Qingxing Yang^1,3^, Nan Wang^1,3^, Xinyu Wang^1^, Jing Feng^1,3✉^, Shuyan Song^1,3✉^ and Hongjie Zhang^1,2,3,4✉^

Correspondence: Jing Feng ([fengj@ciac.ac.cn](mailto:fengj@ciac.ac.cn)), Shuyan Song ([songsy@ciac.ac.cn](mailto:songsy@ciac.ac.cn)) or Hongjie Zhang ([hongjie@ciac.ac.cn](mailto:hongjie@ciac.ac.cn))

*^1^State Key Laboratory of Rare Earth Resource Utilization, Changchun Institute of Applied Chemistry, Chinese Academy of Sciences, Changchun 130022, China*

*^2^State Key Laboratory of Inorganic Synthesis and Preparative Chemistry, College of Chemistry, Jilin University, Changchun 130012, China*

*^3^School of Applied Chemistry and Engineering, University of Science and Technology of China, Hefei 230026, China*

*^4^Department of Chemistry, Tsinghua University, Beijing 100084, China*

**Materials and methods**

**Characterizations**

The powder X-ray diffraction (XRD) patterns were recorded on a Bruker D8 Focus powder X-ray diffractometer using Cu Kα radiation (λ = 1.5418 Å) and operating at a voltage of 40 kV and a current of 40 mA. Structural information was derived from Rietveld refinement using the GSAS Ⅱ software suite. The morphology was recorded by a field emission scanning electron microscope (FE-SEM, S-4800, Hitachi). The element mapping was recorded by a field emission scanning electron microscope (FE-SEM, Sigma300, Zeiss). High-resolution transmission electron microscopes (HRTEM) images were obtained using a FEI Tecnai G2 F20). UV-visible absorption spectra were obtained using a Shimadzu 3600 UV-vis spectrophotometer. The PL and PLE spectra were recorded using a fluorescence spectrometer (Edinburgh Instruments FLS-920) equipped with a xenon lamp as the excitation source. The fluorescent decay curves were obtained from a Lecroy Wave Runner 6100 Digital Oscilloscope (1 GHz) taking a tunable laser (pulse width = 4 ns, gate = 500 ns) and a μF900 lamp as the excitation sources. The element contents were measured using an inductively coupled plasma (ICP) optical emission spectrometer (ICAP6300, Thermo Scientific). The corresponding RL spectra were recorded by FLS920 with an X-ray source (TUB-DATA-1015, Moxtek, 12 W). X-ray imaging was acquired by using a CCD camera. Thermogravimetric analysis (TGA) curve was collected with the Perkin-Elmer STA 6000 instrument. The crystals are heated in the range 40-1000 °C at the heating rate of 10 °C per minute, under N_2_ atmosphere. The measured PL decay curves were fitted with a mono-exponential or bi-exponential decay function, as expressed:$I(t) = I_{0} + {\Sigma_{i}A}_{i}e^{(-t/\tau_{i})}$. The average PL lifetime could be calculated by using $\tau_{ave}={\Sigma_{i}A}_{i}\tau_{i}^{2}/\left（ {\Sigma_{i}A}_{i}\tau_{i} \right）$. *A_i_* represents constant, *τ_i_* is decay time for the exponential component, *I_0_* and *I(t)* are PL intensities at time 0 and t.

**Computational details**

The calculations were carried out using density functional theory with the PBE+U form of generalized gradient approximation functional (GGA).^1^ The Vienna ab-initio simulation package (VASP)^2-5^ was employed. According to previous theory studies,^6-7^ the U correction is 3.2 eV and 6.0 eV for Ce and Eu elements, which is employed in this work. For other elements (Pr, Nd, Sm, Gd), we will employ U values of 6.0 eV.^8^ Due to the limitation of pseudo-potential of Tb-Lu, the electronic structures do not converge and the reasonable structures cannot be obtained. The plane wave energy cutoff was set as 400 eV. The Fermi scheme was employed for electron occupancy with an energy smearing of 0.1 eV. The first Brillouin zone was sampled in the Monkhorst-Pack grid.^9^ The 3×3×3 k-point mesh for the surface calculation. The energy (converged to 1.0 ×10^-6^ eV atom^-1^) and force (converged to 0.01eV Å^-1^) were set as the convergence criterion for geometry optimization. The spin polarization was considered in all calculation.

**Supporting Tables**

**Table S1** The Rietveld refined structural parameters of Cs_3_DyCl_6_, Cs_3_HoCl_6_, Cs_3_TmCl_6_, Cs_3_YbCl_6_, and Cs_3_LuCl_6_ MCs.

| Parameters | Cs_3_DyCl_6_ | Cs_3_HoCl_6_ | Cs_3_TmCl_6_ | Cs_3_YbCl_6_ | Cs_3_LuCl_6_ |
| --- | --- | --- | --- | --- | --- |
| Space group | *C2/c* | *C2/c* | *C2/c* | *C2/c* | *C2/c* |
| *a*(Å) | 26.92(3) | 26.89(1) | 26.83(8) | 26.80(9) | 26.79(0) |
| *b*(Å) | 8.15(7) | 8.13(7) | 8.11(3) | 8.10(5) | 8.09(3) |
| *c*(Å) | 13.12(8) | 13.10(6) | 13.07(7) | 13.05(9) | 13.05(0) |
| *α*(°) | 90.00(0) | 90.00(0) | 90.00(0) | 90.00(0) | 90.00(0) |
| *β*(°) | 99.99(8) | 100.01(4) | 100.05(7) | 100.08(7) | 100.10(5) |
| *γ*(°) | 90.00(0) | 90.00(0) | 90.00(0) | 90.00(0) | 90.00(0) |
| *V*(Å^3^) | 2839.38(6) | 2824.12(5) | 2803.57(6) | 2793.42(4) | 2785.49(4) |
| *R_wp_* | 7.58% | 9.48% | 6.39% | 6.46% | 6.16% |

**Table S2** Inductively coupled plasma optical emission spectrometer (ICP-OES) data of Cs_3_{TbDyHoErTm}_1_Cl_6_ crystals.

| Ln ions in Cs_3_{TbDyHoErTm}_1_Cl_6_ | Feeding ratios | Actual ratios measured by ICP-OES |
| --- | --- | --- |
| Tb/(Tb+Dy+Ho+Er+Tm) | 20% | 22.54% |
| Dy/(Tb+Dy+Ho+Er+Tm) | 20% | 24.92% |
| Ho/(Tb+Dy+Ho+Er+Tm) | 20% | 17.42% |
| Er/(Tb+Dy+Ho+Er+Tm) | 20% | 18.03% |
| Tm/(Tb+Dy+Ho+Er+Tm) | 20% | 17.09% |

**Table S3** PLQYs of Cs_3_LnCl_6_ MCs (Ln = Ce, Pr, Nd, Sm, Eu, Gd, Tb, Dy, Ho, Er, Tm, Yb, Lu).

| Samples | Excitation wavelength | PLQYs |
| --- | --- | --- |
| Cs_3_CeCl_6_ | 340 nm | 53.4% |
| Cs_3_PrCl_6_ | 335 nm | 24.7% |
| Cs_3_NdCl_6_ | 354 nm | 6.6% |
| Cs_3_SmCl_6_ | 380 nm | 2.2% |
| Cs_3_EuCl_6_ | 330 nm | 12.3% |
| Cs_3_GdCl_6_ | 340 nm | 0.2% |
| Cs_3_TbCl_6_ | 280 nm | 90.8% |
| Cs_3_DyCl_6_ | 351 nm | 6.4% |
| Cs_3_HoCl_6_ | 360 nm | 2.2% |
| Cs_3_ErCl_6_ | 380 nm | 0.8% |
| Cs_3_TmCl_6_ | 361 nm | 3.8% |
| Cs_3_YbCl_6_ | 278 nm | 9.4% |
| Cs_3_LuCl_6_ | 340 nm | 0.2% |

**Table S4** Comparison of reported PLQYs with that of this work.

| Ln-based metal halides | PLQY (%) | Reference |
| --- | --- | --- |
| Cs_3_TbCl_6_ | 90.8 | This work |
| Cs_2_NaTbCl_6_ | 56 | 33 |
| Cs_2_NaEuCl_6_ | 35 | 33 |
| Cs_3_CeBr_6_ | ≈90 | 34 |
| CsEuBr_3_ | 68.3 | 35 |
| Cs_2_ZrCl_6_ | 53.0 | 36 |
| Cs_2_PtCl_6_ | 14.10 | 37 |
| CsMnBr_3_ | 11 | 38 |
| Cs_4_MnBi_2_Cl_12_ | 25.7 | 39 |

**Table S5** Comparison of LY and synthesis temperature of the reported Ln-based metal halides scintillators with those of Cs_3_TbCl_6_ MCs in this work.

| Scintillators | LY (photons MeV^-1^) | | Synthesis Temperature (℃) | References |
| --- | --- | --- | --- | --- |
| Cs_3_TbCl_6_ | 51800 | 25 | | This work |
| Cs_2_NaLuCl_6_:Dy | 14344 | 180 | | 22 |
| Cs_2_Na_0.9_Ag_0.1_LuCl_6_:Dy | 8332 | 180 | | 22 |
| Cs_3_TbCl_6_ | 56800 | 450 | | 28 |
| Rb_3_TbCl_6_ | 88800 | 450 | | 28 |
| Cs_2_NaTbCl_6_ | 46600 | 180 | | 33 |
| Cs_2_NaEuCl_6_ | 1250 | 180 | | 33 |
| Cs_2_NaTbCl_6_ | 14800 | 190 | | 42 |
| Cs_2_NaTbCl_6_:Sb | 23500 | 190 | | 42 |

**Table S6** Comparison of X-ray imaging spatial resolution of the reported Ln-based metal halides with that of Cs_3_TbCl_6_@PDMS in this work.

| Scintillators | Spatial resolutions  (lp mm^-1^) | References |
| --- | --- | --- |
| Cs_3_TbCl_6_@PDMS | 12 | This work |
| Cs_2_Na_0.9_Ag_0.1_LuCl_6_:Dy@PET | 11.2 | 22 |
| Cs_3_TbCl_6_ | 5.5 | 27 |
| Cs_3_TbCl_6_:Sb | 9.6 | 27 |
| Rb_3_TbCl_6_@PDMS | 3.9 | 28 |
| Cs_3_TbCl_6_@PDMS | 3.3 | 28 |
| Cs_2_NaTbCl_6_:Sb | 7.9 | 42 |
| Cs_2_NaTbCl_6_ | 4.4 | 42 |

**Supporting Figures**


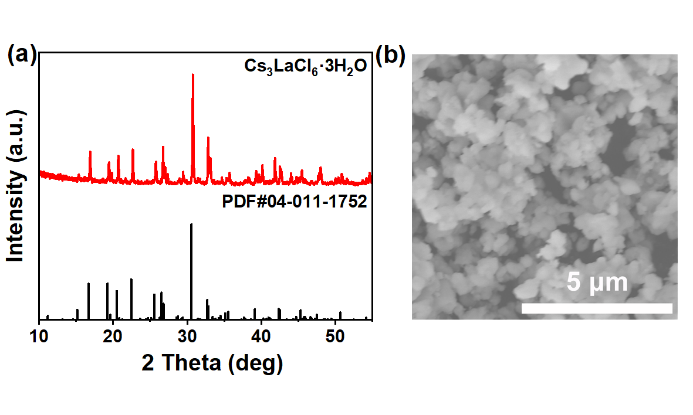
**Fig. S1** (a) Powder XRD pattern and (b) SEM image of Cs_3_LaCl_6_·3H_2_O MCs.

**
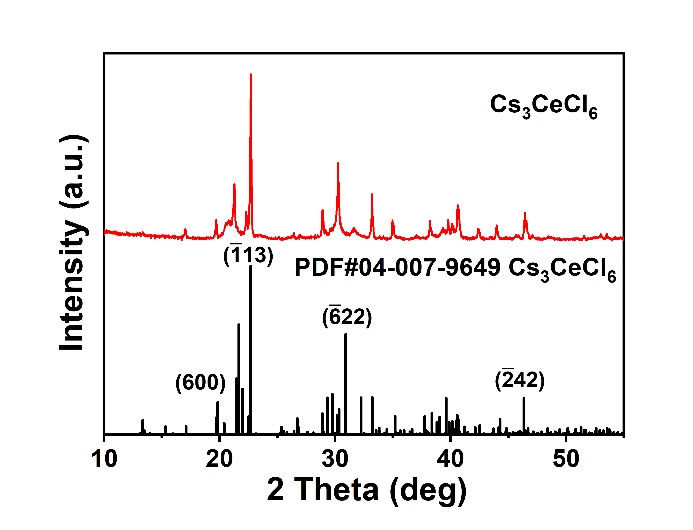
****Fig. S2** Powder XRD pattern and the standard diffraction pattern of Cs_3_CeCl_6_.

**
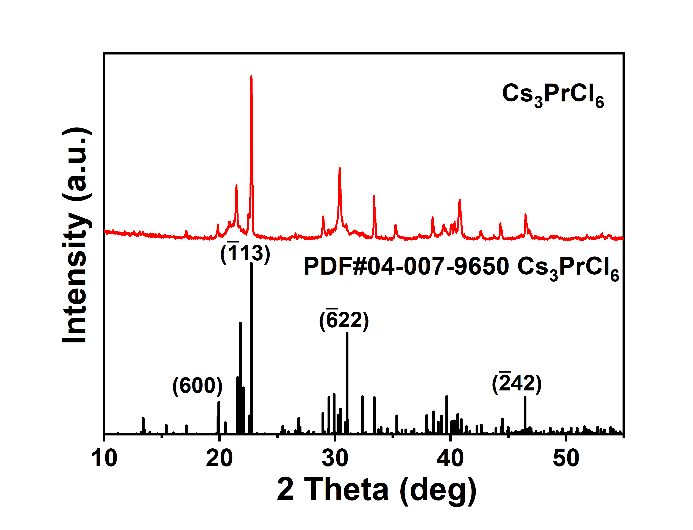
**

**Fig. S3** Powder XRD pattern and the standard diffraction pattern of Cs_3_PrCl_6_.

**
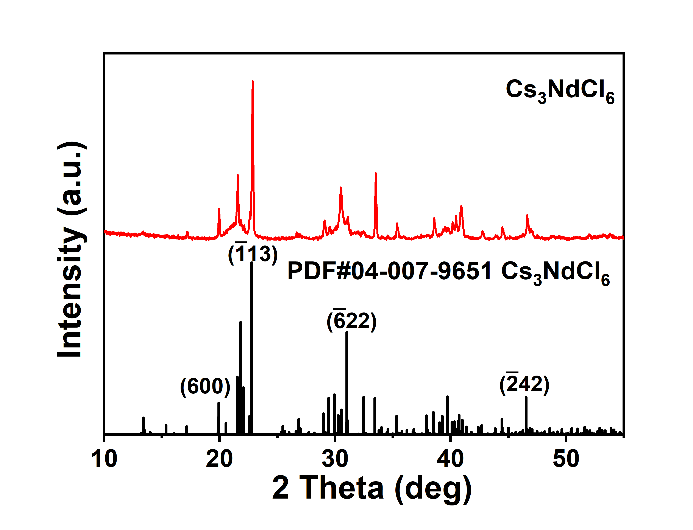
Fig. S4** Powder XRD pattern and the standard diffraction pattern of Cs_3_NdCl_6_.

**
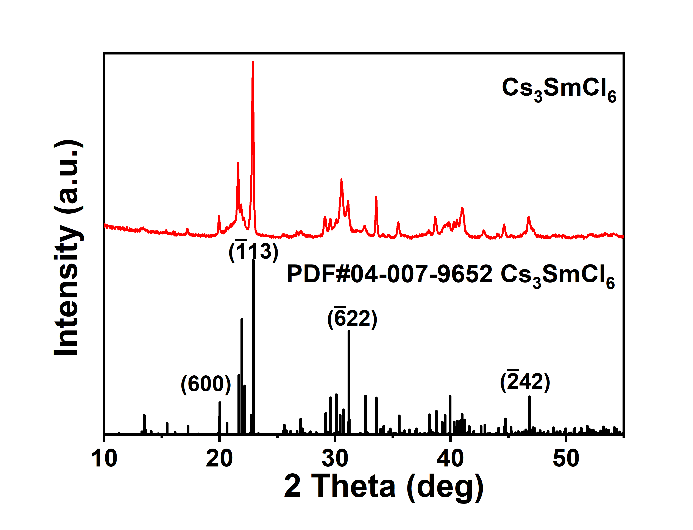
**

**Fig. S5** Powder XRD pattern and the standard diffraction pattern of Cs_3_SmCl_6_.

**
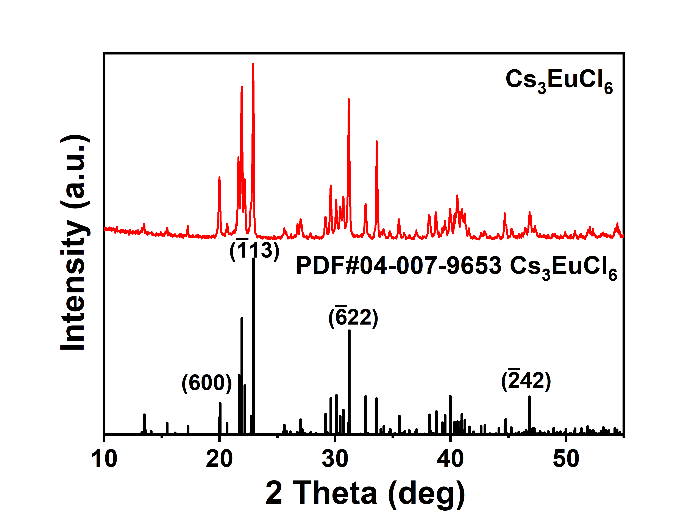
Fig. S6** Powder XRD pattern and the standard diffraction pattern of Cs_3_EuCl_6_.

**
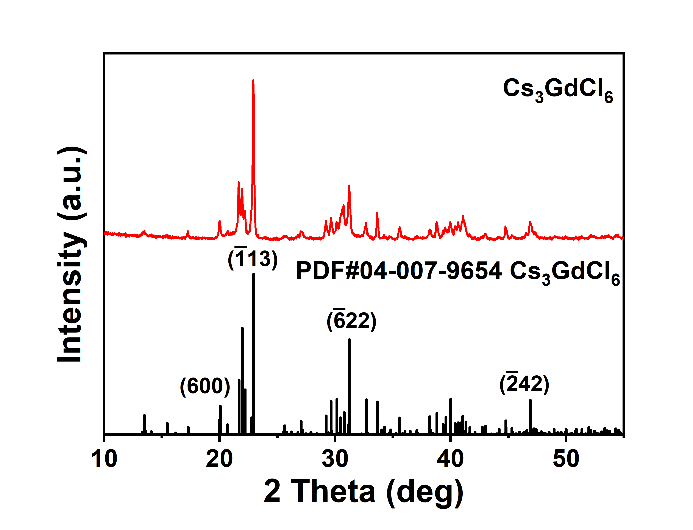
**

**Fig. S7** Powder XRD pattern and the standard diffraction pattern of Cs_3_GdCl_6_.

**
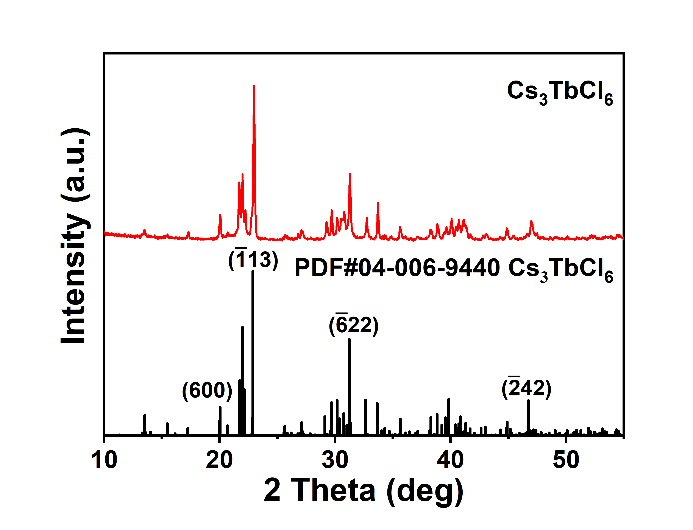
**

**Fig. S8** Powder XRD pattern and the standard diffraction pattern of Cs_3_TbCl_6_.

**
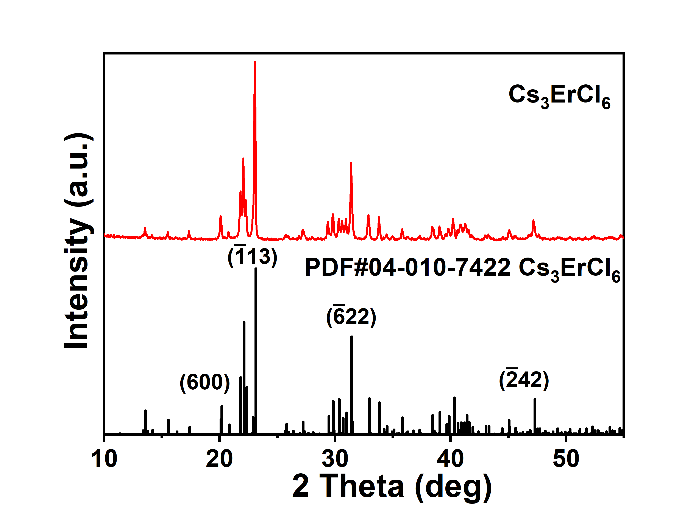
**

**Fig. S9** Powder XRD pattern and the standard diffraction pattern of Cs_3_ErCl_6_.


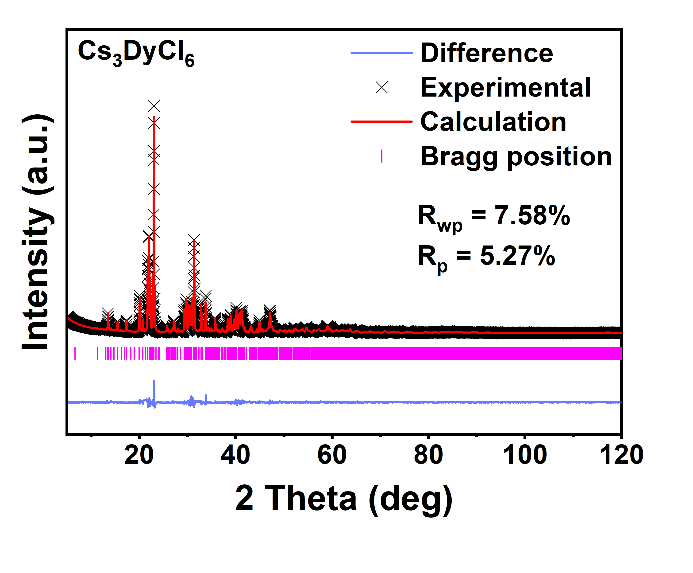
**Fig. S10** The Rietveld refinement X-ray diffraction plot of Cs_3_DyCl_6_ MCs.


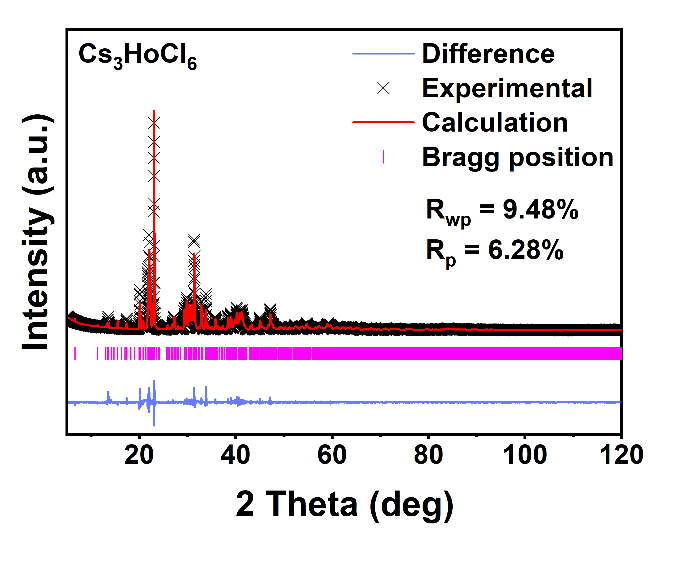


**Fig. S11** The Rietveld refinement X-ray diffraction plot of Cs_3_HoCl_6_ MCs.


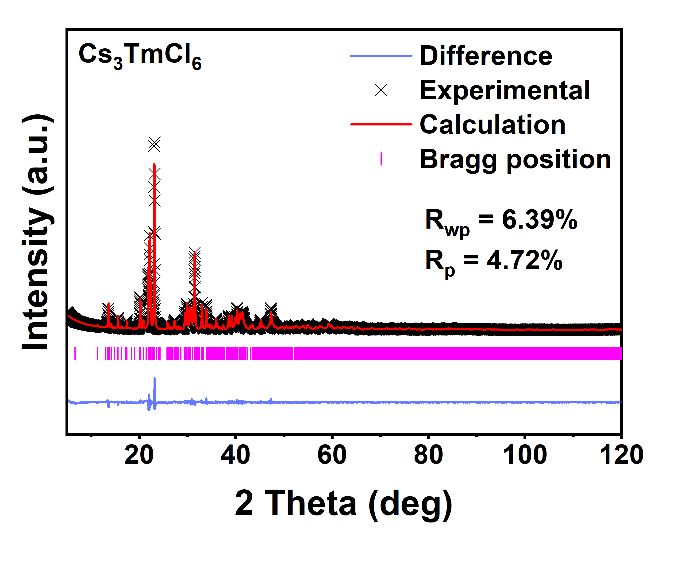


**Fig. S12** The Rietveld refinement X-ray diffraction plot of Cs_3_TmCl_6_ MCs.


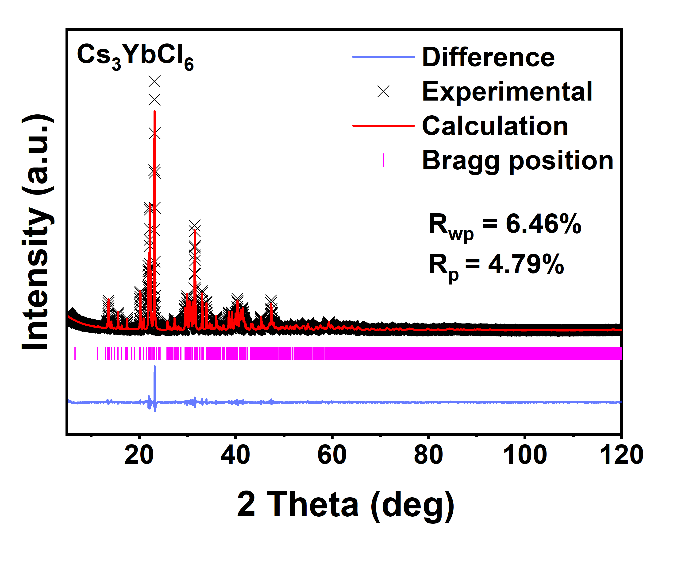


**Fig. S13** The Rietveld refinement X-ray diffraction plot of Cs_3_YbCl_6_ MCs.


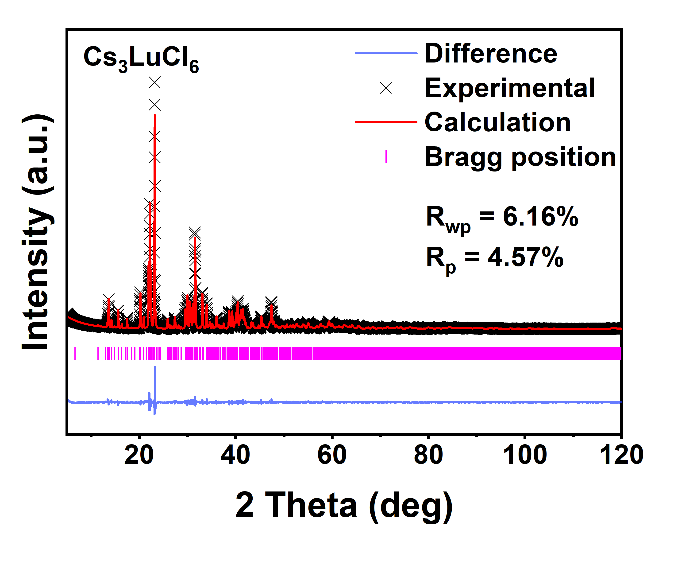


**Fig. S14** The Rietveld refinement X-ray diffraction plot of Cs_3_LuCl_6_ MCs.


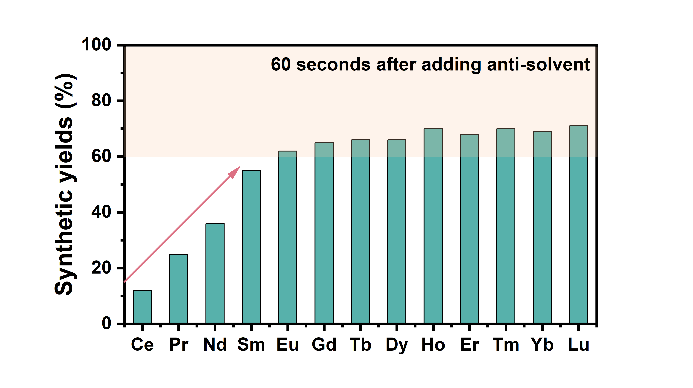
**Fig. S15** The synthetic yields of Cs_3_LnCl_6_ MCs after adding anti-solvent for 60 seconds.


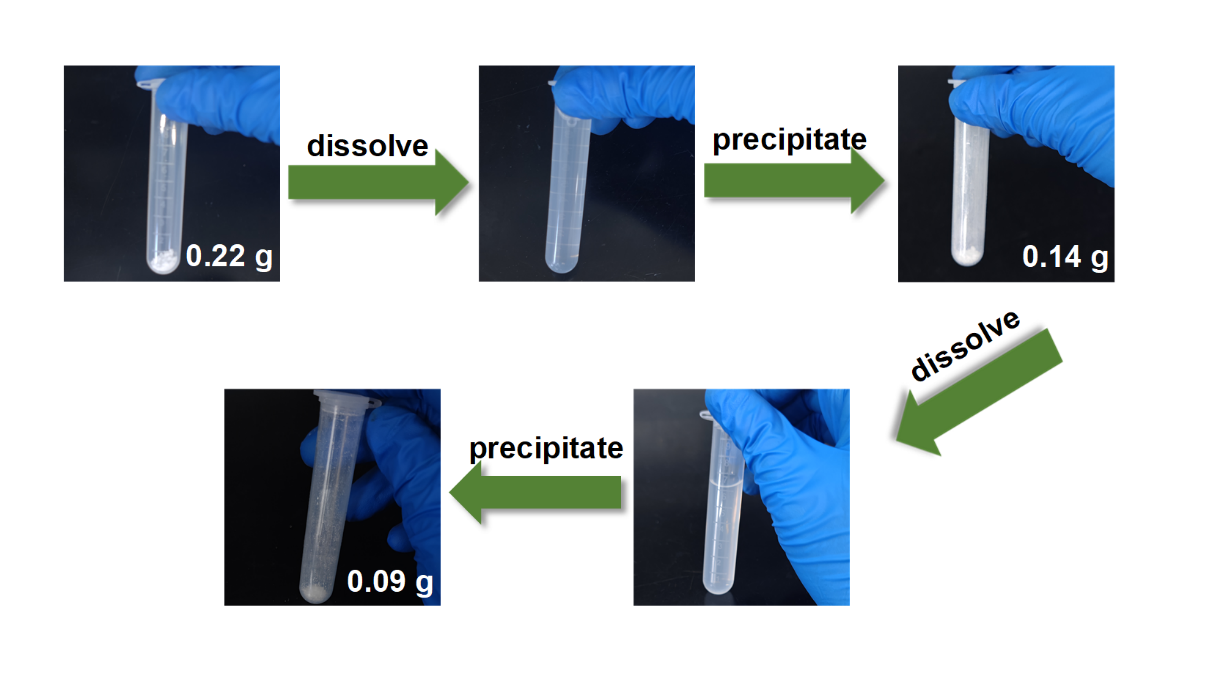
**Fig. S16** The reversible synthesis of Cs_3_TbCl_6_ MCs.


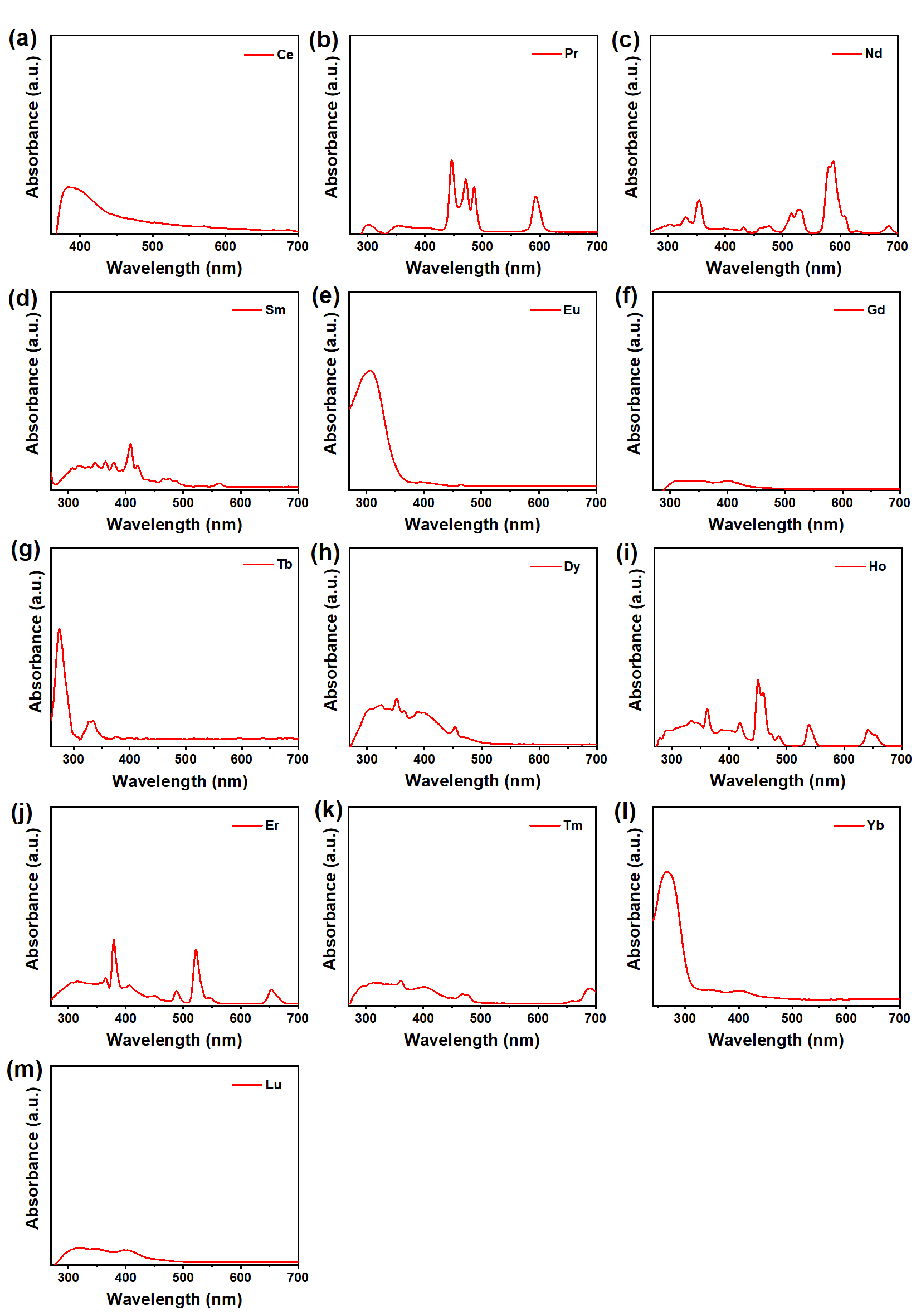
**Fig. S17** The UV-Vis absorption spectra of Cs_3_LnCl_6_ MCs (Ln = Ce, Pr, Nd, Sm, Eu, Gd, Tb, Dy, Ho, Er, Tm, Yb, Lu).

**
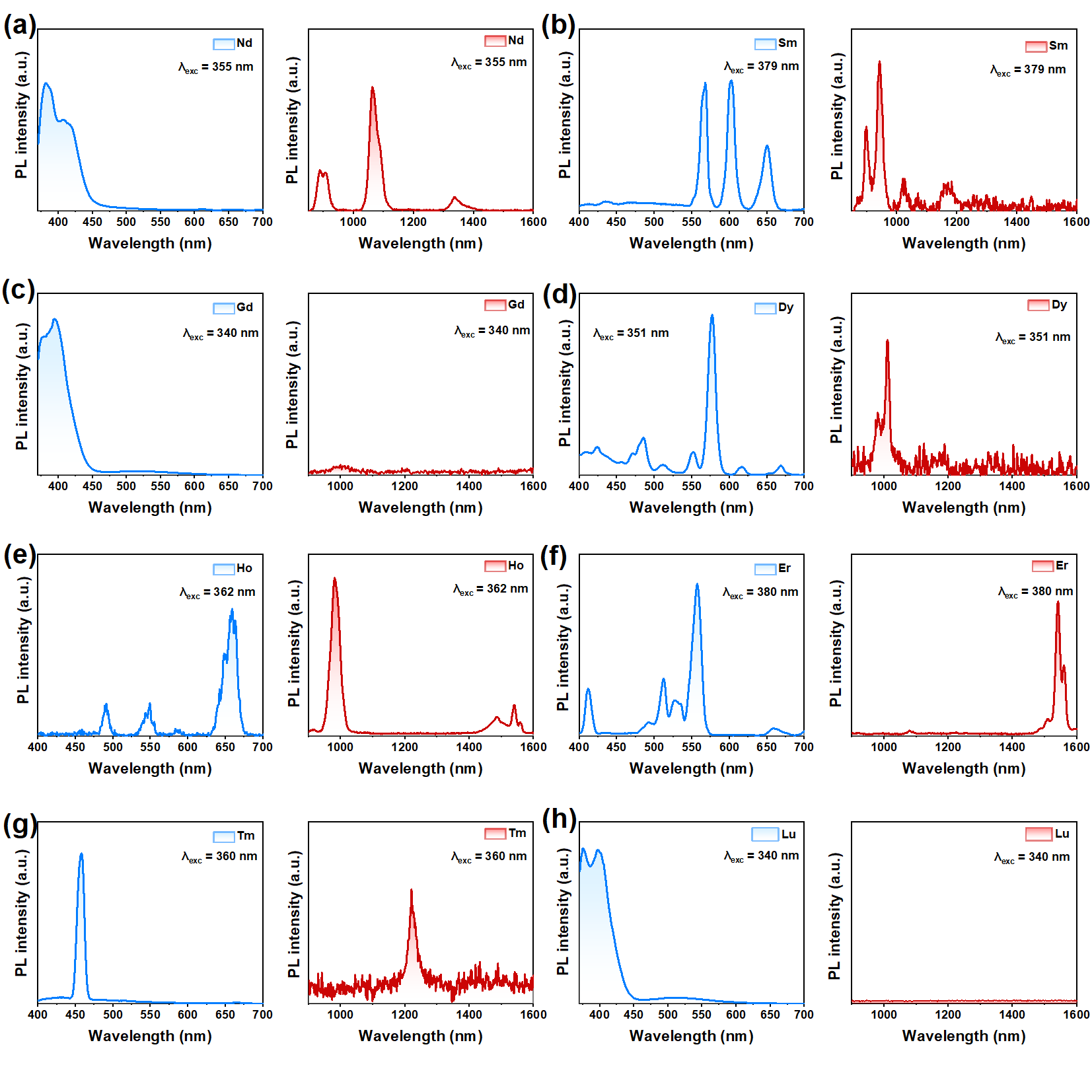
Fig. S18** PL spectra of Cs_3_LnCl_6_ MCs (Ln = Nd, Sm, Gd, Dy, Ho, Er, Tm, Lu) in the visible region (left) and NIR region (right).

**
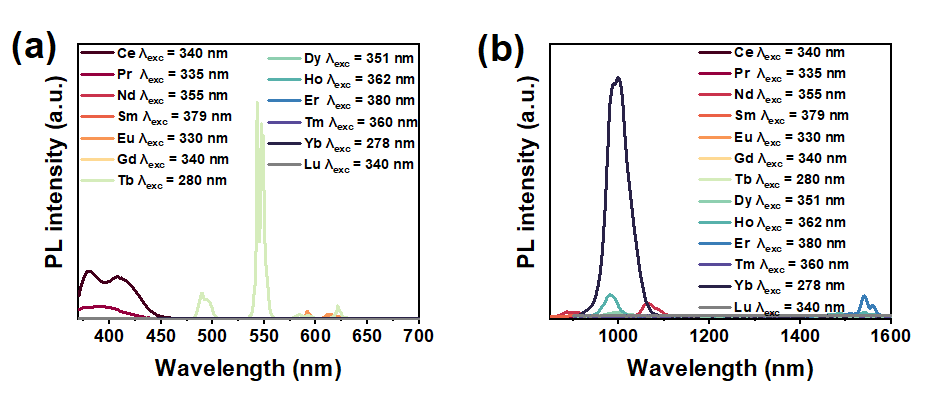
Fig. S19** PL spectra of Cs_3_LnCl_6_ MCs (Ln = Ce, Pr, Nd, Sm, Eu, Gd, Tb, Dy, Ho, Er, Tm, Yb, Lu) in the (a) visible region and (b) NIR region.

**
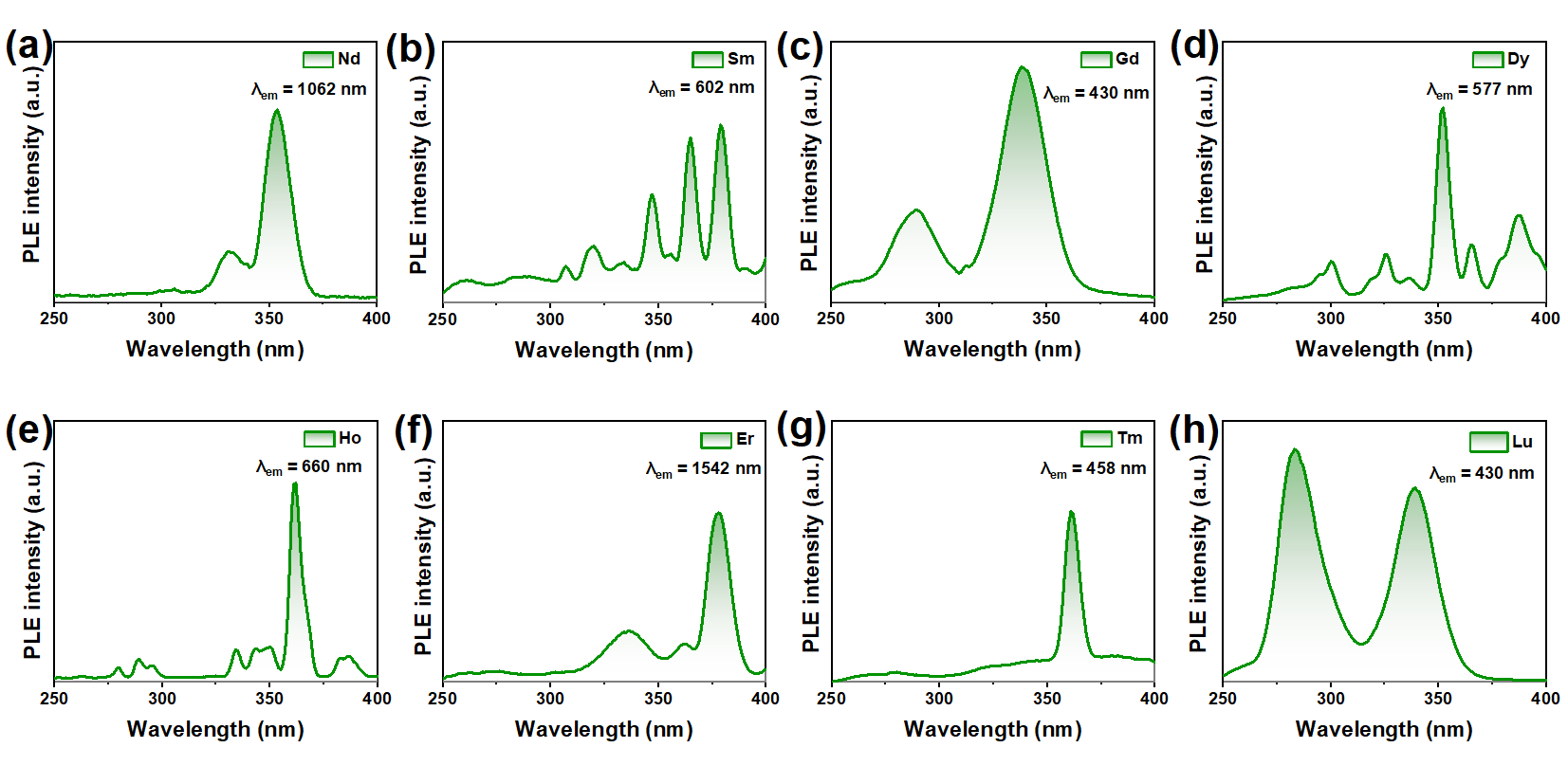
Fig. S20** PLE spectra of Cs_3_LnCl_6_ MCs (Ln = Nd, Sm, Gd, Dy, Ho, Er, Tm, Lu).


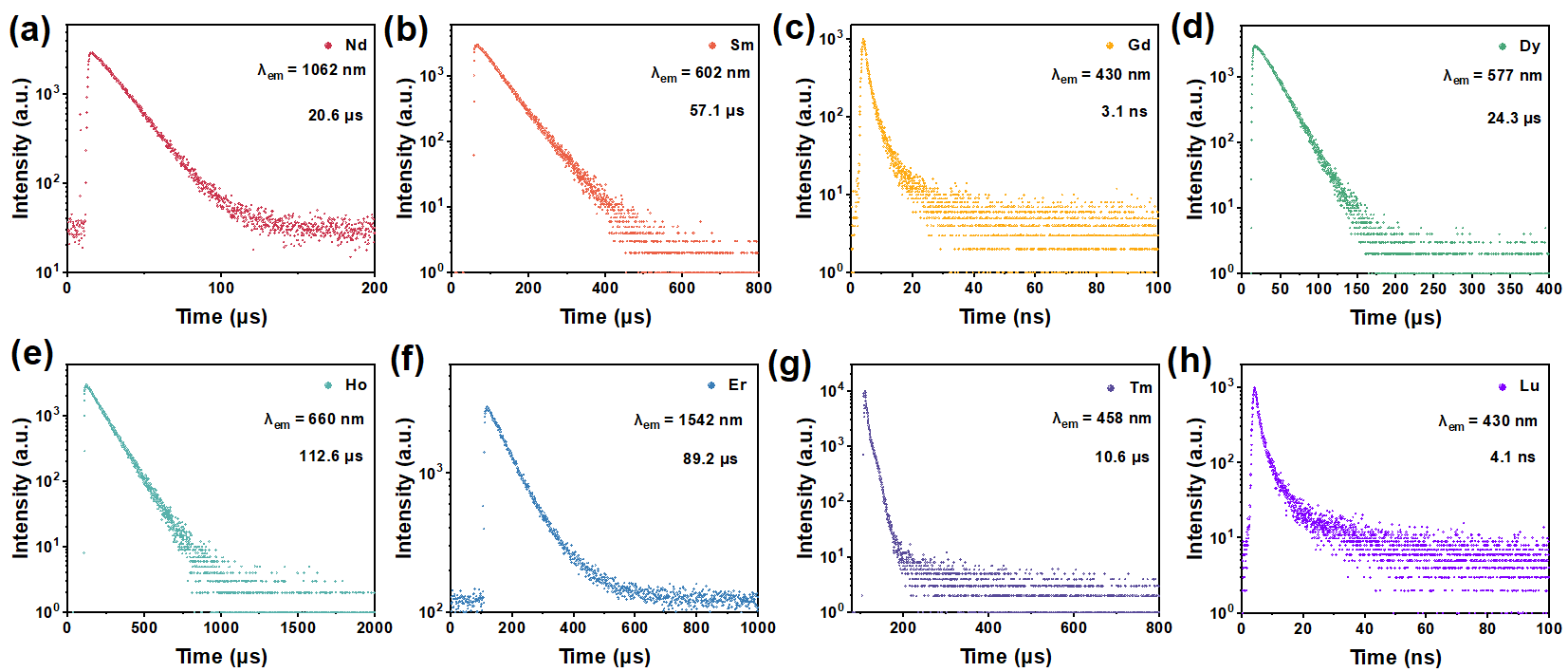


**Fig. S21** PL decay curves of Cs_3_LnCl_6_ MCs (Ln = Nd, Sm, Gd, Dy, Ho, Er, Tm, Lu).


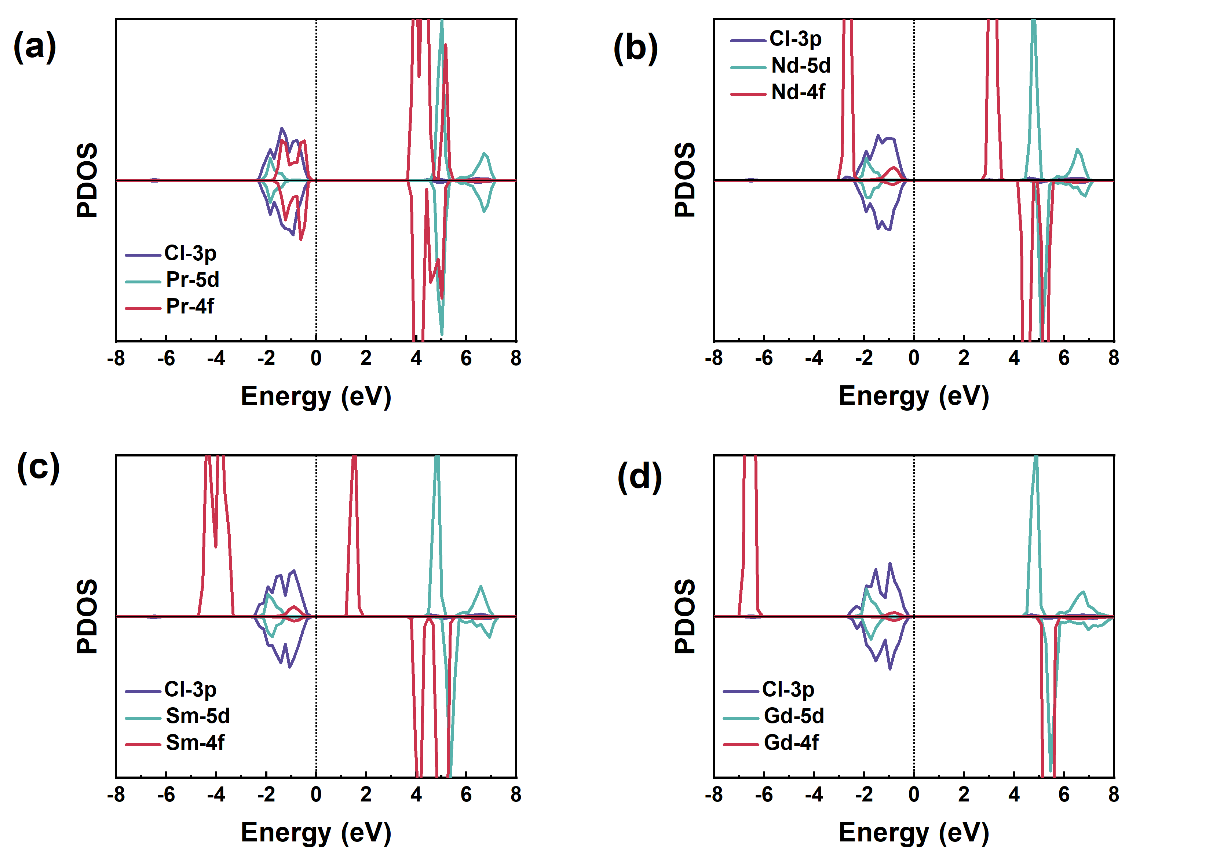
**Fig. S22** The PDOS of Cs_3_LnCl_6_ (Ln = Pr, Nd, Sm, Gd).


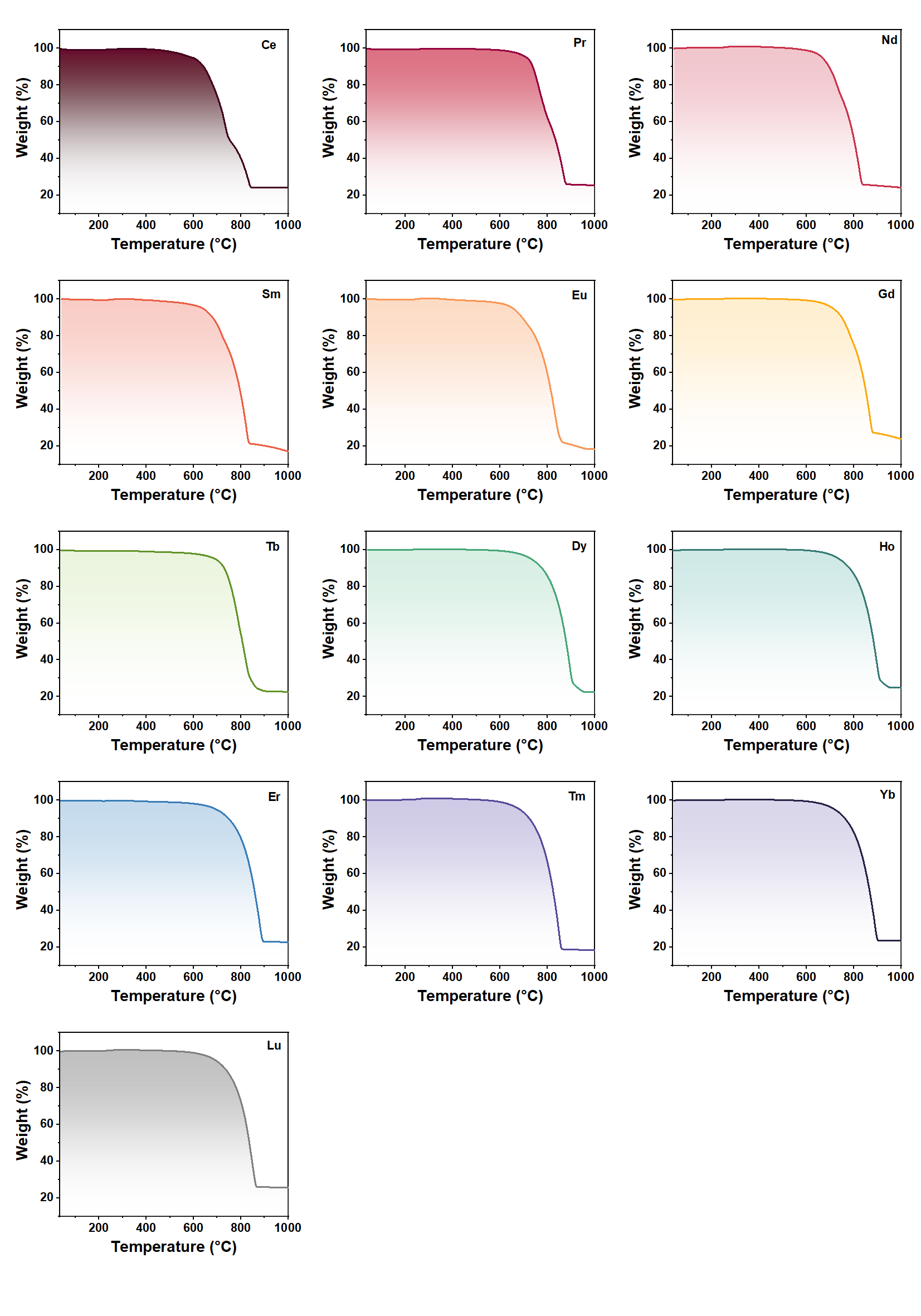
**Fig. S23** TG curves of Cs_3_LnCl_6_ MCs (Ln = Ce, Pr, Nd, Sm, Eu, Gd, Tb, Dy, Ho, Er, Tm, Yb, Lu).

**
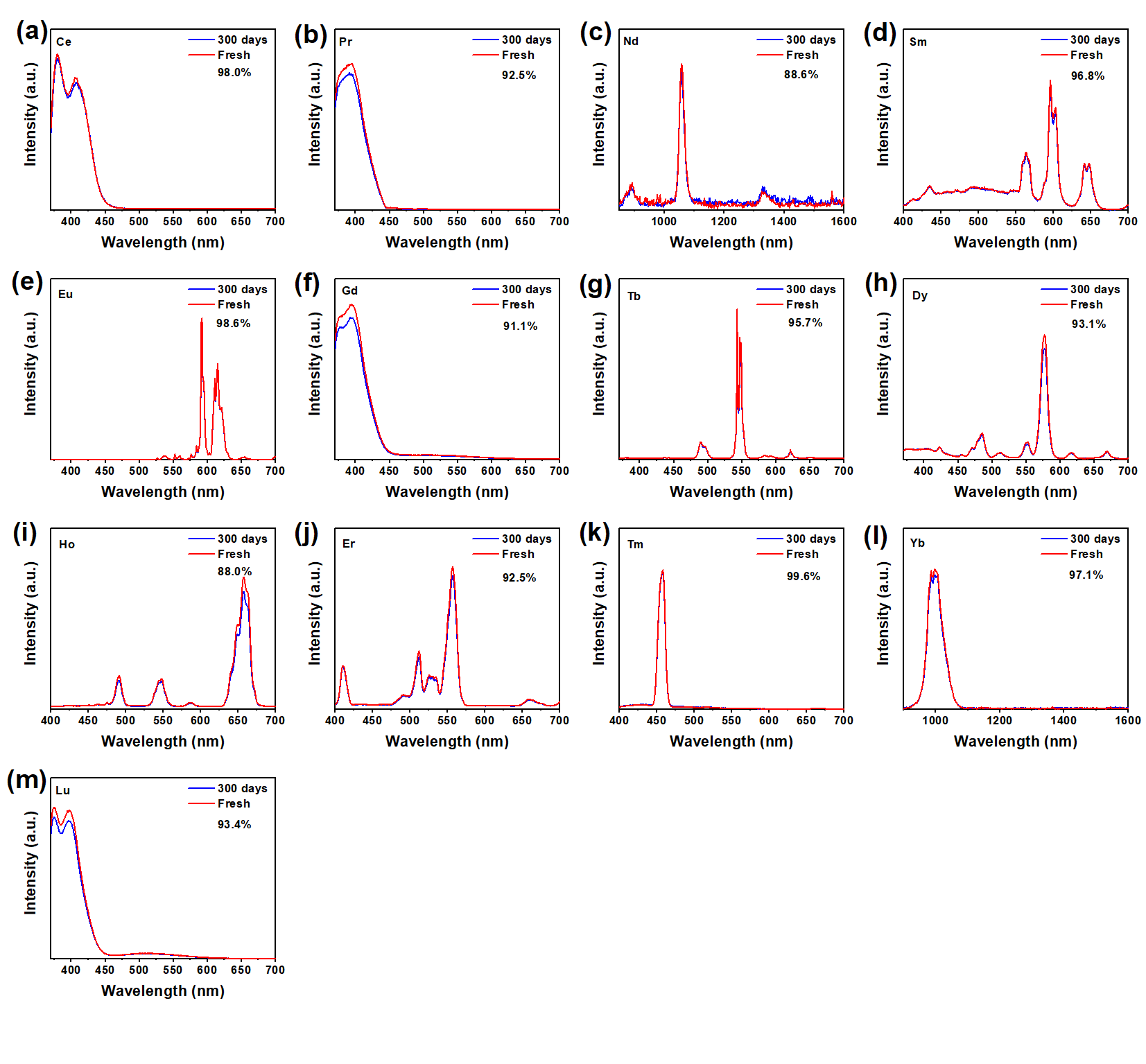
**

**Fig. S24** PL spectra of fresh Cs_3_LnCl_6_ MCs and Cs_3_LnCl_6_ MCs (Ln = Ce, Pr, Nd, Sm, Eu, Gd, Tb, Dy, Ho, Er, Tm, Yb, Lu) after storing for 300 days.


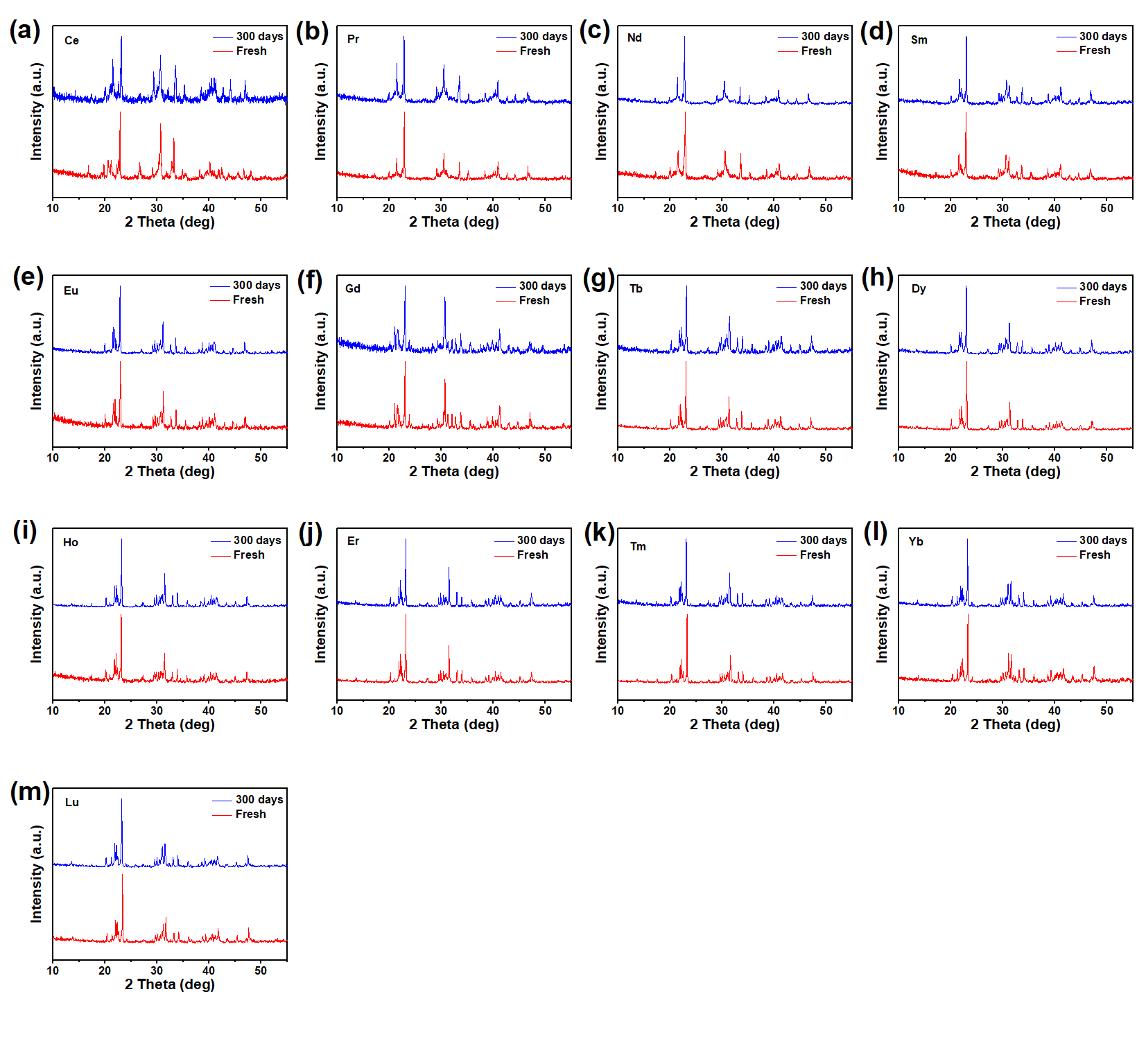
**Fig. S25** Powder XRD patterns of fresh Cs_3_LnCl_6_ MCs and Cs_3_LnCl_6_ MCs (Ln = Ce, Pr, Nd, Sm, Eu, Gd, Tb, Dy, Ho, Er, Tm, Yb, Lu) after storing for 300 days.


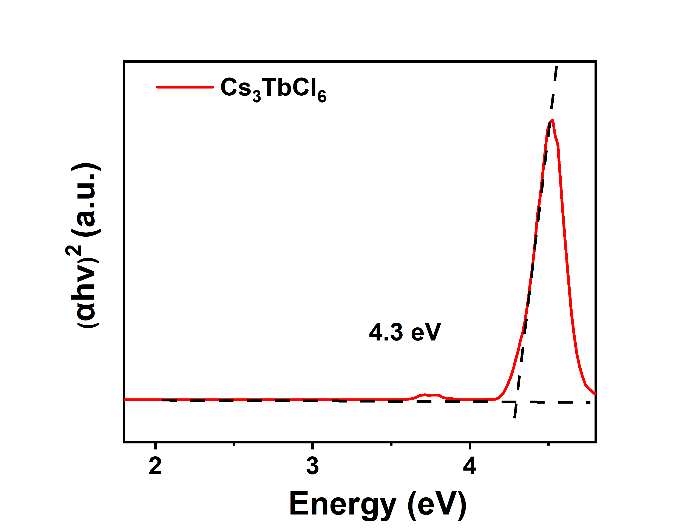
**Fig. S26** Tauc plot of Cs_3_TbCl_6_ MCs.


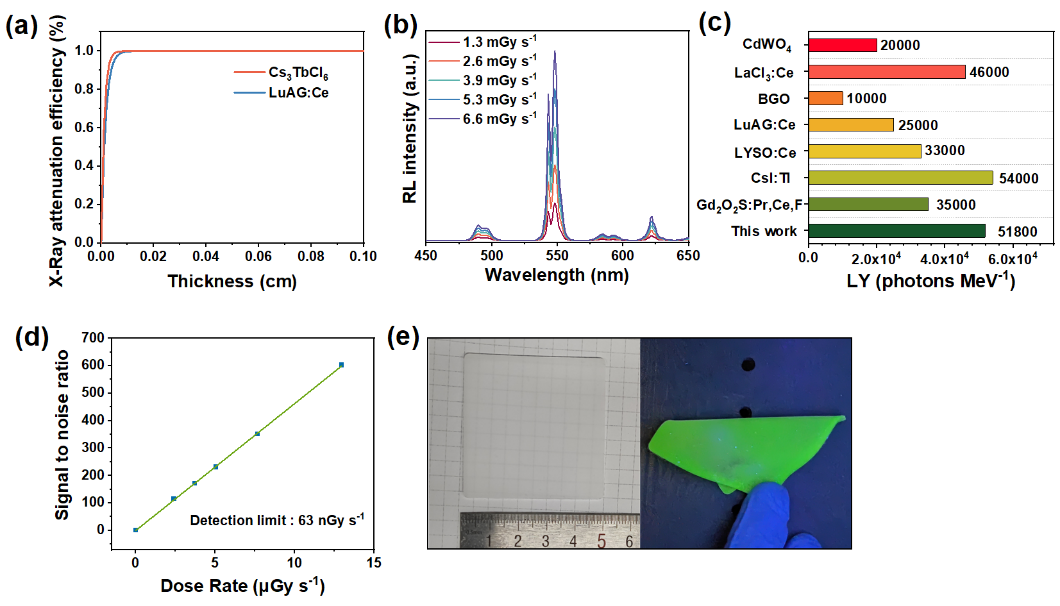
**Fig. S27** (a) X-ray attenuation efficiency of Cs_3_TbCl_6_ MCs and the typical scintillator LuAG:Ce as a function of the thickness (X-ray photon energy of 8.5 keV). (b) RL spectra of Cs_3_TbCl_6_ as a function of X-ray dose rate. (c) Comparison of LY between as-prepared Cs_3_TbCl_6_ and some commercial scintillators. (d) Linear relationship between signal to noise ratio value and X-ray dose rate. (e) Photographs of large-sized and transparent Cs_3_TbCl_6_@PDSM thin film under visible light (left) and UV light (right).

**References**

1. Perdew, J. P., Burke, K. & Ernzerhof, M. Generalized Gradient Approximation Made Simple. *Phys. Rev. Lett.* **77**, 3865-3868 (1996).
2. Kresse, G. & Furthmüller, J. Efficiency of ab-initio total energy calculations for metals and semiconductors using a plane-wave basis set. *Comp. Mater. Sci.* **6**, 15-50 (1996).
3. Kresse, G. & Hafner, J. Ab initiomolecular dynamics for liquid metals. *Phys. Rev. B* **47**, 558-561 (1993).
4. Kresse, G. & Hafner, J. Ab initiomolecular-dynamics simulation of the liquid-metal-amorphous-semiconductor transition in germanium. *Phys. Rev. B* **49**, 14251-14269 (1994).
5. Kresse, G. & Furthmüller, J. Efficient iterative schemes for ab initio total-energy calculations using a plane-wave basis set. *Phys. Rev. B* **54**, 11169-11186 (1996).
6. Chen, J., Guo, Y., Chen, B., Zheng, W. & Wang, F. Ultrafast and Multicolor Luminescence Switching in a Lanthanide-Based Hydrochromic Perovskite. *J. Am. Chem. Soc.* **144**, 22295-22301 (2022).
7. Wang, L. et al. Exploration of Nontoxic Cs_3_CeBr_6_ for Violet Light-Emitting Diodes. *ACS Energy Lett.* **6**, 4245-4254 (2021).
8. Pang, M. *et al.* First-principles study on the crystal, electronic structure and mechanical properties of hexagonal Al_3_RE (RE = La, Ce, Pr, Nd, Sm, Gd) intermetallic compounds. *Solid State Commun.* **151**, 1135-1140 (2011).
9. Monkhorst, H. J. & Pack, J. D. Special points for Brillouin-zone integrations. *Phys. Rev. B* **13**, 5188-5192 (1976).
